# Supplementary material for: Modeling transfer of vaginal microbiota from mother to infant in early life
Source: eLife. 2021 Jan 15;10:e57051. doi: 10.7554/eLife.57051 (PMC7810462; doi:10.7554/eLife.57051)
Supplement: Supplementary file 1. [file elife-57051-supp1.zip › FullAnalysis.html]

Modelling transfer of vaginal microbiota from mother to infant (in early life)


# Modelling transfer of vaginal microbiota from mother to infant (in early life)

- 0 - Prep
  - 0.1 - Load libraries
  - 0.2 Download main data
  - 0.3 Download precalculated data
  - 0.4 - Overview of samples, read counts and observed richness
- 1 - Vaginal microbiome
  - 1.1 - Data prep
  - 1.2 - Observed vaginal ASV’s:
  - 1.3 - Community State Types
    - 1.3.1 - Define Community State Types
      - 1.3.1.1 - Find optimal number of clusters
      - 1.3.1.2 - Create CSTs
    - 1.3.2 - CST composition and stability
      - 1.3.2.1 - Prepare data
      - 1.3.2.2 - Describe CST composition and stability
      - 1.3.2.3 - Stability between w24 and w36 (Permutational)
        - 1.3.2.3.1 - Perform permutation calculations
        - 1.3.2.3.2 - CST stability results
  - 1.4 Beta diversity of vaginal
    - 1.4.1 - NMDS plot of vaginal samples
    - 1.4.2 - Statistical test of beta diversity
- 2 - Infant Samples
  - 2.1 - Delivery mode
  - 2.2 - Airway microbiome
    - 2.2.1 - Subset airway samples
    - 2.2.2 - Dominant airway taxa and overall richness
      - 2.2.2.1 - tables
      - 2.2.2.2 - Plot
    - 2.2.3 - Airway alpha diversity
    - 2.2.4 - Airway beta diversity
      - 2.2.4.1 - Preparation
      - 2.2.4.2 - Plots and statistics
    - 2.2.5 - Create figure 2 illustrating airway microbiomes
  - 2.3 - Fecal microbiome
    - 2.3.1 - Subset fecal samples
    - 2.3.2 - Dominant fecal taxa and overall richness
      - 2.3.2.1 - tables
      - 2.3.2.2 - Plot
    - 2.3.3 - Fecal alpha diversity
    - 2.3.4 - Fecal beta diversity
      - 2.3.4.1 - Preparation
      - 2.3.4.2 - Plots and statistics
    - 2.3.5 - Create figure 3 illustrating fecal microbiomes
- 3 - Transfer
  - 3.1 - Preparation
    - 3.1.1 - Calculation of ALL individual ASV models
    - 3.1.2 - Permutational inference calculation
      - 3.1.2.1 - Calculations
      - 3.1.2.2 - Format output
      - 3.1.2.3 - Overview of testable ASVs
      - 3.1.2.4 - ASVs with significant transfer odds
    - 3.1.3 - Plot ASV transfer odds
      - 3.1.3.1 - Figure 4-figure supplement 1 - vaginal delivery
      - 3.1.3.2 - Figure 4-figure supplement 2 - sectio delivery
      - 3.1.3.3 - Figure 4-figure supplement 3 - In labor sectio delivery
      - 3.1.3.4 - Figure 4-figure supplement 4 - Scheduled sectio delivery
      - 3.1.3.5 - Figure 4-figure supplement statistics
  - 3.2 - Weighted Odds Ratio
    - 3.2.1 - OVERALL ratio between positive and negative odds
      - 3.2.1.1 - WTR Calculation
      - 3.2.1.2 - WTR tables and figures
    - 3.2.2 - WTR at order level
      - 3.2.2.1 - Comparing vaginal to sectio delivery
        - 3.2.2.1.1 - Calculations
        - 3.2.2.1.2 - Output
      - 3.2.2.2 - Comparing vaginal to scheduled and in labor sectio delivery
        - 3.2.2.2.1 - Calculations
        - 3.2.2.2.2 - Output
  - 3.3 - Transfer of mothers dominant ASV
    - 3.3.1 - Calculations
    - 3.3.2 - Output
  - 3.4 - Phylogenetic tree with transfer odds

# 0 - Prep

Data for this project is from the COPSAC2010 cohort of 711 children / mother pairs. In this project we include vaginal samples (week 24 and week 36), airway samples (1 week, 1 month, and 3 months), and fecal samples (1 week, 1 month, and 1 year) for all mother-child dyads which include a week 36 vaginal sample (665 mothers and 651 children). We describe the vaginal microbiome development from mid pregnancy (week 24) to late pregnancy (week 36), and the transfer to the airways and gut of the children in the first year of life. A special focus is on the differences between transfer to vaginal and sectio born children. All tables created as part of this analysis can also be found in the *Source data 1* file **Supplementary\_results\_tables.xlsx**

## 0.1 - Load libraries

## 0.2 Download main data

**COPSACbirthmicrobiome\_ASV.RData** contains the phyloseq object that is used for all subsequent analysis and with this the whole analysis can be easily replicated

## 0.3 Download precalculated data

To reduce the computational requirements during this analysis, we have precalculated the most computer intensive parts and if these are downloaded the calculations can be skipped. This means that you can either run this code chunk or all following code chunks which are currently set not to be evaluated.

## 0.4 - Overview of samples, read counts and observed richness

Phyloseq object used:

```
## phyloseq-class experiment-level object
## otu_table()   OTU Table:         [ 15514 taxa and 4756 samples ]
## sample_data() Sample Data:       [ 4756 samples by 4 sample variables ]
## tax_table()   Taxonomy Table:    [ 15514 taxa by 10 taxonomic ranks ]
## phy_tree()    Phylogenetic Tree: [ 15514 tips and 15356 internal nodes ]
```

Sample count per time and type

| Time | Samples |
| --- | --- |
| **Vaginal** | |
| Week\_24 | 657 |
| Week\_36 | 665 |
| **Fecal** | |
| One\_week | 533 |
| One\_month | 575 |
| One\_year | 580 |
| **Airway** | |
| One\_week | 526 |
| One\_month | 606 |
| Three\_months | 614 |

ASVs observed per compartment

| Compartment | ASVs |
| --- | --- |
| Vaginal | 3287 |
| Fecal | 6818 |
| Airways | 7500 |

Histogram of sequencing depth of study samples by type and time point

Summary stats for compartment

|  | Count | | | | | Observed Richness | | | | | | |
| --- | --- | --- | --- | --- | --- | --- | --- | --- | --- | --- | --- | --- |
| Type | Median | Mean | SD | Q25 | Q75 | Median | Mean | SD | Min | Max | Q25 | Q75 |
| Vaginal | 50121.0 | 53875.3 | 23442.5 | 38329.8 | 64631.5 | 20 | 25.0 | 18.8 | 2 | 167 | 12 | 32 |
| Fecal | 50014.5 | 55171.8 | 38967.9 | 30132.2 | 75199.0 | 37 | 45.2 | 27.3 | 6 | 298 | 26 | 60 |
| Airway | 51232.0 | 54496.2 | 35433.9 | 29259.2 | 73810.5 | 26 | 28.8 | 15.6 | 1 | 231 | 19 | 36 |

Summary stats for compartment/time point

|  |  | Count | | | | | Observed Richness | | | | | | |
| --- | --- | --- | --- | --- | --- | --- | --- | --- | --- | --- | --- | --- | --- |
| Type | Time | Median | Mean | SD | Q25 | Q75 | Median | Mean | SD | Min | Max | Q25 | Q75 |
| Vaginal | Week\_24 | 49918.0 | 53074.2 | 23023.6 | 37689.0 | 63701.0 | 20 | 25.3 | 17.9 | 2 | 125 | 13 | 32.0 |
| Vaginal | Week\_36 | 50341.0 | 54666.7 | 23840.0 | 38704.0 | 65933.0 | 19 | 24.7 | 19.7 | 2 | 167 | 11 | 31.0 |
| Fecal | One\_week | 53540.0 | 59559.5 | 44625.8 | 23051.0 | 91744.0 | 29 | 32.1 | 19.1 | 8 | 298 | 22 | 37.0 |
| Fecal | One\_month | 51612.0 | 57232.7 | 41400.8 | 33938.5 | 75985.5 | 29 | 31.9 | 15.0 | 6 | 175 | 22 | 39.0 |
| Fecal | One\_year | 47810.0 | 49096.4 | 28920.9 | 31266.0 | 63858.5 | 67 | 70.5 | 25.3 | 12 | 175 | 54 | 84.2 |
| Airway | One\_week | 45304.5 | 51252.8 | 35794.3 | 23772.5 | 72282.2 | 20 | 22.6 | 13.3 | 2 | 231 | 16 | 26.0 |
| Airway | One\_month | 58918.0 | 60030.7 | 35014.2 | 40285.5 | 77725.5 | 27 | 29.1 | 16.2 | 3 | 226 | 20 | 35.0 |
| Airway | Three\_months | 46566.0 | 51812.3 | 34933.3 | 27468.2 | 70207.2 | 33 | 34.0 | 14.9 | 1 | 114 | 24 | 44.0 |

# 1 - Vaginal microbiome

## 1.1 - Data prep

**vag\_taxglm.RData** contains phyloseq objects with vaginal samples at phylum, genus and ASV level for both read counts and relative abundances, as well as a rarefied (2000 reads/sample) at ASV level. **OrdinationRes.RData** contains weighted UniFrac distances and jensen-Shannon divergence, as well as NMDS ordinations of both

## 1.2 - Observed vaginal ASV’s:

The distribution of the vaginal reads are here summarized on phylum, genus and individual ASV level.

Count of phyla, genera, and ASV in vaginal samples

| Included | Phylum | Genus | ASV |
| --- | --- | --- | --- |
| All | 28 | 463 | 3287 |
| > 0.01% | 16 | 193 | 958 |
| > 0.1% | 10 | 94 | 420 |
| > 1% | 8 | 41 | 173 |

Average abundance according to phylum

| Kingdom | Phylum | taxprc |
| --- | --- | --- |
| Bacteria | Firmicutes | 85.2 |
| Bacteria | Actinobacteria | 12.0 |
| Bacteria | Proteobacteria | 1.5 |
| Bacteria | Bacteroidetes | 0.8 |
| Bacteria | Fusobacteria | 0.2 |
| Bacteria | Tenericutes | 0.1 |

Average abundance according to genus

| Kingdom | Phylum | Class | Order | Family | Genus | taxprc |
| --- | --- | --- | --- | --- | --- | --- |
| Bacteria | Firmicutes | Bacilli | Lactobacillales | Lactobacillaceae | Lactobacillus | 81.1 |
| Bacteria | Actinobacteria | Actinobacteria | Bifidobacteriales | Bifidobacteriaceae | Gardnerella | 9.0 |
| Bacteria | Actinobacteria | Actinobacteria | Bifidobacteriales | Bifidobacteriaceae | Bifidobacterium | 1.4 |
| Bacteria | Actinobacteria | Coriobacteriia | Coriobacteriales | Atopobiaceae | Atopobium | 1.3 |
| Bacteria | Proteobacteria | Gammaproteobacteria | Enterobacteriales | Enterobacteriaceae | Escherichia\_Shigella | 1.2 |
| Bacteria | Firmicutes | Negativicutes | Selenomonadales | Veillonellaceae | Megasphaera | 0.9 |

Average abundance according to ASV

| Kingdom | Phylum | Class | Order | Family | Genus | Species | name | taxprc |
| --- | --- | --- | --- | --- | --- | --- | --- | --- |
| Bacteria | Firmicutes | Bacilli | Lactobacillales | Lactobacillaceae | Lactobacillus | Genus\_Lactobacillus | Genus\_Lactobacillus\_323 | 31.5 |
| Bacteria | Firmicutes | Bacilli | Lactobacillales | Lactobacillaceae | Lactobacillus | Genus\_Lactobacillus | Genus\_Lactobacillus\_139 | 29.5 |
| Bacteria | Firmicutes | Bacilli | Lactobacillales | Lactobacillaceae | Lactobacillus | Lactobacillus\_gasseri | Lactobacillus\_gasseri\_37 | 10.4 |
| Bacteria | Actinobacteria | Actinobacteria | Bifidobacteriales | Bifidobacteriaceae | Gardnerella | Genus\_Gardnerella | Genus\_Gardnerella\_40 | 4.6 |
| Bacteria | Firmicutes | Bacilli | Lactobacillales | Lactobacillaceae | Lactobacillus | Genus\_Lactobacillus | Genus\_Lactobacillus\_268 | 4.5 |
| Bacteria | Actinobacteria | Actinobacteria | Bifidobacteriales | Bifidobacteriaceae | Gardnerella | Genus\_Gardnerella | Genus\_Gardnerella\_20 | 3.8 |

## 1.3 - Community State Types

The vaginal microbiome is *not* a smooth continoum, but a set of very well defined clusters, here refered to as Community State Types (CST), and a few less well defined clusters. These are identified by clustering of *all* the samples based on Jensen Shannon Divergence as beta diversity measure. Partitioning around medoids clustering is then performed for a range of possible clusters and the optimal number defined based on various cluster statistics.

### 1.3.1 - Define Community State Types

#### 1.3.1.1 - Find optimal number of clusters

Barplots of clustering statistics for 3-9 clusters

Based on the Pearson version of Hubert’s gamma coefficient (pearsongamma), average silhouette width (avg.silwidth) and the Calinski and Harabasz index (ch) 5 or 6 clusters is optimal. Considering the Dunn2 index we consider *6* clusters to be optimal. These are refered to as community state types I to V, with IV being split into IV-a and IV-b.

#### 1.3.1.2 - Create CSTs

The ASVs for the top ASVs in each CST are written to **BlastData.xlsx**, Blast results and identified species for each ASV have then been added to this file externally.

**phyX\_cst.RData** contains the updated phyloseq objects for all samples (phyX), vaginal samples (vagX), and rarefied vaginal samples (vagX.r)

### 1.3.2 - CST composition and stability

#### 1.3.2.1 - Prepare data

**CommunityStateTypes.RData** contains the necessary data regarding dominant taxa and alpha diversity of the CSTs

#### 1.3.2.2 - Describe CST composition and stability

Observed richness and shannon diversity index (SDI) for each CST

|  |  | Observed Richness | | Shannon Diversity index | |
| --- | --- | --- | --- | --- | --- |
| CST | Samples (n) | Mean | SD | Mean | SD |
| CST\_I | 479 | 13.44 | 10.68 | 0.48 | 0.47 |
| CST\_II | 172 | 18.31 | 15.68 | 0.90 | 0.61 |
| CST\_III | 446 | 12.37 | 9.85 | 0.55 | 0.52 |
| CST\_IV\_a | 86 | 22.21 | 15.79 | 1.30 | 0.71 |
| CST\_IV\_b | 68 | 19.82 | 14.53 | 1.23 | 0.59 |
| CST\_V | 71 | 14.32 | 11.44 | 0.89 | 0.47 |

**Anova statistical test of alpha diversity by CST**  
Overall differences in observed richness between CSTs

```
## Analysis of Variance Table
## 
## Response: Observed
##             Df Sum Sq Mean Sq F value    Pr(>F)    
## CST          5  12075 2414.94  17.245 < 2.2e-16 ***
## Residuals 1316 184286  140.04                      
## ---
## Signif. codes:  0 '***' 0.001 '**' 0.01 '*' 0.05 '.' 0.1 ' ' 1
```

Multiple comparison of differences in observed richness between all pairs of CSTs

```
##   Tukey multiple comparisons of means
##     95% family-wise confidence level
## 
## Fit: aov(formula = Observed ~ CST, data = cst.alpha)
## 
## $CST
##                         diff         lwr        upr     p adj
## CST_II-CST_I       4.8734524   1.8713975  7.8755074 0.0000581
## CST_III-CST_I     -1.0727880  -3.2950528  1.1494767 0.7405034
## CST_IV_a-CST_I     8.7688013   4.8136112 12.7239914 0.0000000
## CST_IV_b-CST_I     6.3830284   2.0064816 10.7595751 0.0004797
## CST_V-CST_I        0.8834426  -3.4113731  5.1782584 0.9919023
## CST_III-CST_II    -5.9462405  -8.9774971 -2.9149838 0.0000004
## CST_IV_a-CST_II    3.8953488  -0.5648737  8.3555714 0.1269766
## CST_IV_b-CST_II    1.5095759  -3.3282146  6.3473665 0.9488339
## CST_V-CST_II      -3.9900098  -8.7539891  0.7739694 0.1603017
## CST_IV_a-CST_III   9.8415893   5.8641892 13.8189894 0.0000000
## CST_IV_b-CST_III   7.4558164   3.0591877 11.8524452 0.0000215
## CST_V-CST_III      1.9562307  -2.3590474  6.2715088 0.7885822
## CST_IV_b-CST_IV_a -2.3857729  -7.8662302  3.0946844 0.8158579
## CST_V-CST_IV_a    -7.8853587 -13.3007712 -2.4699461 0.0004936
## CST_V-CST_IV_b    -5.4995857 -11.2299719  0.2308004 0.0684618
```

Overall differences in Shannon diversity index between CSTs

```
## Analysis of Variance Table
## 
## Response: Shannon
##             Df Sum Sq Mean Sq F value    Pr(>F)    
## CST          5  92.71 18.5411   66.18 < 2.2e-16 ***
## Residuals 1316 368.69  0.2802                      
## ---
## Signif. codes:  0 '***' 0.001 '**' 0.01 '*' 0.05 '.' 0.1 ' ' 1
```

Multiple comparison of differences in Shannon diversity index between all pairs of CSTs

```
##   Tukey multiple comparisons of means
##     95% family-wise confidence level
## 
## Fit: aov(formula = Shannon ~ CST, data = cst.alpha)
## 
## $CST
##                          diff         lwr        upr     p adj
## CST_II-CST_I       0.42730580  0.29302794  0.5615836 0.0000000
## CST_III-CST_I      0.07762367 -0.02177523  0.1770226 0.2251923
## CST_IV_a-CST_I     0.82552360  0.64861330  1.0024339 0.0000000
## CST_IV_b-CST_I     0.75856486  0.56280786  0.9543219 0.0000000
## CST_V-CST_I        0.40995745  0.21785616  0.6020587 0.0000000
## CST_III-CST_II    -0.34968213 -0.48526613 -0.2140981 0.0000000
## CST_IV_a-CST_II    0.39821780  0.19871809  0.5977175 0.0000002
## CST_IV_b-CST_II    0.33125907  0.11487125  0.5476469 0.0001950
## CST_V-CST_II      -0.01734834 -0.23043468  0.1957380 0.9999077
## CST_IV_a-CST_III   0.74789993  0.56999621  0.9258037 0.0000000
## CST_IV_b-CST_III   0.68094120  0.48428595  0.8775964 0.0000000
## CST_V-CST_III      0.33233379  0.13931724  0.5253503 0.0000149
## CST_IV_b-CST_IV_a -0.06695873 -0.31209217  0.1781747 0.9709838
## CST_V-CST_IV_a    -0.41556614 -0.65779021 -0.1733421 0.0000162
## CST_V-CST_IV_b    -0.34860741 -0.60491982 -0.0922950 0.0015199
```

Top five Phylums / Genus / ASVs for each CST

|  | Phylum | | Genus | | ASV | |
| --- | --- | --- | --- | --- | --- | --- |
| Rank | Phylum | Abundance (%) | Genus | Abundance (%) | ASV | Abundance (%) |
| **CST I** | | | | | | |
| 1 | Firmicutes | 94.95 | Lactobacillus | 91.94 | Genus\_Lactobacillus\_323 | 84.35 |
| 2 | Actinobacteria | 3.32 | Bifidobacterium | 1.84 | Genus\_Lactobacillus\_139 | 2.60 |
| 3 | Proteobacteria | 1.25 | Enterococcus | 1.11 | Genus\_Bifidobacterium\_60 | 1.54 |
| 4 | Bacteroidetes | 0.27 | Escherichia\_Shigella | 1.02 | Genus\_Enterococcus\_34 | 1.15 |
| 5 | Tenericutes | 0.12 | Gardnerella | 0.96 | Genus\_Escherichia\_Shigella\_101 | 0.85 |
| **CST II** | | | | | | |
| 1 | Firmicutes | 84.91 | Lactobacillus | 80.21 | Lactobacillus\_gasseri\_37 | 70.65 |
| 2 | Actinobacteria | 11.54 | Gardnerella | 6.85 | Genus\_Gardnerella\_40 | 4.04 |
| 3 | Proteobacteria | 1.94 | Bifidobacterium | 2.69 | Genus\_Gardnerella\_20 | 2.49 |
| 4 | Bacteroidetes | 1.31 | Escherichia\_Shigella | 1.60 | Genus\_Bifidobacterium\_60 | 2.44 |
| 5 | Epsilonbacteraeota | 0.17 | Atopobium | 1.36 | Lactobacillus\_gasseri\_47 | 2.24 |
| **CST III** | | | | | | |
| 1 | Firmicutes | 92.34 | Lactobacillus | 89.23 | Genus\_Lactobacillus\_139 | 81.17 |
| 2 | Actinobacteria | 5.03 | Gardnerella | 3.67 | Genus\_Lactobacillus\_268 | 2.55 |
| 3 | Proteobacteria | 1.69 | Escherichia\_Shigella | 1.20 | Genus\_Lactobacillus\_323 | 2.03 |
| 4 | Bacteroidetes | 0.48 | Megasphaera | 0.93 | Genus\_Gardnerella\_20 | 1.55 |
| 5 | Fusobacteria | 0.34 | Bifidobacterium | 0.85 | Genus\_Gardnerella\_40 | 1.53 |
| **CST IV a** | | | | | | |
| 1 | Actinobacteria | 62.40 | Gardnerella | 58.26 | Genus\_Gardnerella\_40 | 51.17 |
| 2 | Firmicutes | 32.20 | Lactobacillus | 19.53 | Genus\_Gardnerella\_20 | 6.17 |
| 3 | Bacteroidetes | 2.84 | Megasphaera | 5.52 | Genus\_Megasphaera\_22 | 5.33 |
| 4 | Fusobacteria | 0.88 | Atopobium | 3.57 | Genus\_Lactobacillus\_139 | 4.82 |
| 5 | Proteobacteria | 0.82 | Prevotella | 2.54 | Lactobacillus\_gasseri\_37 | 3.96 |
| **CST IV b** | | | | | | |
| 1 | Actinobacteria | 61.31 | Gardnerella | 46.75 | Genus\_Gardnerella\_20 | 45.28 |
| 2 | Firmicutes | 32.48 | Lactobacillus | 24.44 | Genus\_Atopobium\_36 | 12.89 |
| 3 | Proteobacteria | 2.76 | Atopobium | 12.64 | Lactobacillus\_gasseri\_37 | 12.84 |
| 4 | Bacteroidetes | 2.42 | Escherichia\_Shigella | 2.55 | Genus\_Lactobacillus\_323 | 2.64 |
| 5 | Fusobacteria | 0.59 | Prevotella | 2.26 | Genus\_Escherichia\_Shigella\_101 | 2.59 |
| **CST V** | | | | | | |
| 1 | Firmicutes | 89.33 | Lactobacillus | 87.62 | Genus\_Lactobacillus\_268 | 60.76 |
| 2 | Actinobacteria | 7.93 | Gardnerella | 6.09 | Genus\_Lactobacillus\_139 | 20.64 |
| 3 | Proteobacteria | 1.41 | Atopobium | 1.62 | Genus\_Gardnerella\_20 | 2.69 |
| 4 | Bacteroidetes | 1.00 | Prevotella | 0.95 | Genus\_Gardnerella\_7 | 2.01 |
| 5 | Epsilonbacteraeota | 0.20 | Escherichia\_Shigella | 0.89 | Genus\_Lactobacillus\_265 | 1.71 |

Figure 1: Vaginal community state type (CST). (A) Boxplot of top Amplicon sequence variant (ASV) abundance for each CST (including two most abundant ASVs from each CST), (B) boxplot of observed richness by CST, (c) boxplot of Shannon diversity index by CST, and (D) alluvial plot showing the CST at weeks 24–36 for each woman. All plots are colored by the CST.

#### 1.3.2.3 - Stability between w24 and w36 (Permutational)

In order to test the stability between time points, a permutation procedure is used. Here, the distance based on individual women from week 24 to week 36 are compared with random assignments of pairs.

##### 1.3.2.3.1 - Perform permutation calculations

**Stability\_w24\_to\_w36\_permresults.RData** contains the permutation results for CST stability

##### 1.3.2.3.2 - CST stability results

overall stability of CSTs from week 24 to week 36

| Women | In same (%) | Changed (%) |
| --- | --- | --- |
| 657 | 562 (85.5) | 95 (14.5) |

CST dependent stability from week 24 to week 36

| CST | Time point (week) | Women | In same (%) |
| --- | --- | --- | --- |
| CST\_I | 24 | 239 | 221 (92.5) |
| CST\_I | 36 | 237 | 221 (93.2) |
| CST\_II | 24 | 86 | 70 (81.4) |
| CST\_II | 36 | 84 | 70 (83.3) |
| CST\_III | 24 | 213 | 194 (91.1) |
| CST\_III | 36 | 232 | 194 (83.6) |
| CST\_IV\_a | 24 | 45 | 30 (66.7) |
| CST\_IV\_a | 36 | 41 | 30 (73.2) |
| CST\_IV\_b | 24 | 35 | 21 (60.0) |
| CST\_IV\_b | 36 | 31 | 21 (67.7) |
| CST\_V | 24 | 39 | 26 (66.7) |
| CST\_V | 36 | 32 | 26 (81.2) |

Pearson’s Chi-squared test of CST stability

```
## 
##  Pearson's Chi-squared test
## 
## data:  df.cst.24$w36 by df.cst.24$CST 
## X-squared = 58.4038, df = 5, p-value = 2.596e-11
## alternative hypothesis: true difference in probabilities is not equal to 0 
## sample estimates:
##    proba in group CST_I   proba in group CST_II  proba in group CST_III 
##               0.9246862               0.8139535               0.9107981 
## proba in group CST_IV_a proba in group CST_IV_b    proba in group CST_V 
##               0.6666667               0.6000000               0.6666667 
## 
##         Pairwise comparisons using Pearson's Chi-squared tests with Yates' continuity correction
## 
##              CST_I  CST_II   CST_III CST_IV_a CST_IV_b
## CST_II   1.617e-02       -         -        -        -
## CST_III  7.758e-01 0.05140         -        -        -
## CST_IV_a 1.293e-05 0.14324 9.690e-05        -        -
## CST_IV_b 1.699e-06 0.04716 1.293e-05   0.7758        -
## CST_V    2.903e-05 0.15598 1.968e-04   1.0000   0.7758
## 
## P value adjustment method: fdr
```

Distance from week 24 to week 36 samples within pairs (internal) and to randomly paired women (external, # permutations = 2500)

|  |  | Median distance | |  |  |
| --- | --- | --- | --- | --- | --- |
| Distance metric | pairs | internal | external | Ratio\* | p-value |
| jsd | 657 | 0.03090 | 0.63491 | 20.54648 | 4e-04 |
| wuf | 657 | 0.00258 | 0.00974 | 3.77142 | 4e-04 |
|  |
| --- |
| \* external / internal distance |

Stability between week 24 and week 36 dependent on week 24 CST

| CST | Samples (n) | Median distance |
| --- | --- | --- |
| **Jensen-Shannon divergence** | | |
| CST\_I | 239 | 0.0232 |
| CST\_II | 86 | 0.0639 |
| CST\_III | 213 | 0.0220 |
| CST\_IV\_a | 45 | 0.0763 |
| CST\_IV\_b | 35 | 0.0927 |
| CST\_V | 39 | 0.0680 |
| **Weighted UniFrac distances** | | |
| CST\_I | 239 | 0.0016 |
| CST\_II | 86 | 0.0160 |
| CST\_III | 213 | 0.0014 |
| CST\_IV\_a | 45 | 0.0057 |
| CST\_IV\_b | 35 | 0.0095 |
| CST\_V | 39 | 0.0039 |

Barplot of median Jensen-Shannon divergence (jsd) and Weighted UniFrac distance between each woman’s vaginal samples by CSTs

Inference (kruskal walis) for differences in stability between week 24 and week 36 dependent on week 24 CST

| Distance metric | Rank sum statistic | p-value | degrees of freedom |
| --- | --- | --- | --- |
| jsd | 49.6 | 1.66e-09 | 5 |
| wuf | 143 | 4.02e-29 | 5 |

The statistics indicate that stability depends on CST.

## 1.4 Beta diversity of vaginal

Here PCoA plots show the distirbution of the samples based on CST and beta diversity metric. Clearly, some of the CST are more well defined than others. E.g. CST\_IV\_b and CST\_IV\_c are all over the place. This is further confirmed by the statistical analysis of the betadispertion wich clearly show that CST IVa and CST IVb are significantly more dispersed than the other CSTs, with CST I and CST II being the tightest clusters

### 1.4.1 - NMDS plot of vaginal samples

Figure 1-figure supplement 1: Non-metric multidimenionsal scaling plot based on Jensen–Shannon divergence; samples colored by community state type (CST), and gray lines connect samples from the individual women

### 1.4.2 - Statistical test of beta diversity

**Betadisper test differences in dispersion of each cluster**  
Overall differences between CSTs

```
## Analysis of Variance Table
## 
## Response: Distances
##             Df  Sum Sq Mean Sq F value    Pr(>F)    
## Groups       5  3.3709 0.67418  43.072 < 2.2e-16 ***
## Residuals 1316 20.5984 0.01565                      
## ---
## Signif. codes:  0 '***' 0.001 '**' 0.01 '*' 0.05 '.' 0.1 ' ' 1
```

Multiple comparison of differences between all pairs of CSTs

```
##   Tukey multiple comparisons of means
##     95% family-wise confidence level
## 
## Fit: aov(formula = distances ~ group, data = df)
## 
## $group
##                          diff          lwr          upr     p adj
## CST_II-CST_I       0.06275195  0.031013266  0.094490639 0.0000003
## CST_III-CST_I      0.01589486 -0.007599637  0.039389352 0.3836737
## CST_IV_a-CST_I     0.14997156  0.108156027  0.191787100 0.0000000
## CST_IV_b-CST_I     0.16691971  0.120649452  0.213189960 0.0000000
## CST_V-CST_I        0.08410557  0.038699403  0.129511738 0.0000022
## CST_III-CST_II    -0.04685709 -0.078904511 -0.014809679 0.0004588
## CST_IV_a-CST_II    0.08721961  0.040064710  0.134374512 0.0000023
## CST_IV_b-CST_II    0.10416775  0.053021083  0.155314425 0.0000001
## CST_V-CST_II       0.02135362 -0.029012696  0.071719933 0.8321304
## CST_IV_a-CST_III   0.13407671  0.092026358  0.176127054 0.0000000
## CST_IV_b-CST_III   0.15102485  0.104542281  0.197507416 0.0000000
## CST_V-CST_III      0.06821071  0.022588211  0.113833216 0.0003058
## CST_IV_b-CST_IV_a  0.01694814 -0.040993007  0.074889293 0.9610337
## CST_V-CST_IV_a    -0.06586599 -0.123119468 -0.008612516 0.0134341
## CST_V-CST_IV_b    -0.08281414 -0.143397613 -0.022230657 0.0014063
```

Boxplot of betadispertion per CST

PERMANOVA of Time point using Jensen-Shannon divergence

```
## 
## Call:
## adonis(formula = vag.jsd ~ Time, data = df) 
## 
## Permutation: free
## Number of permutations: 999
## 
## Terms added sequentially (first to last)
## 
##             Df SumsOfSqs  MeanSqs F.Model      R2 Pr(>F)
## Time         1     0.046 0.046152 0.30907 0.00023  0.868
## Residuals 1320   197.113 0.149328         0.99977       
## Total     1321   197.159                  1.00000
```

PERMANOVA of CST using Jensen-Shannon divergence

```
## 
## Call:
## adonis(formula = vag.jsd ~ CST, data = df) 
## 
## Permutation: free
## Number of permutations: 999
## 
## Terms added sequentially (first to last)
## 
##             Df SumsOfSqs MeanSqs F.Model      R2 Pr(>F)    
## CST          5   158.310  31.662  1072.5 0.80296  0.001 ***
## Residuals 1316    38.849   0.030         0.19704           
## Total     1321   197.159                 1.00000           
## ---
## Signif. codes:  0 '***' 0.001 '**' 0.01 '*' 0.05 '.' 0.1 ' ' 1
```

# 2 - Infant Samples

## 2.1 - Delivery mode

Summary of delivery mode

|  | Children | |
| --- | --- | --- |
| Delivery | n | % |
| Vaginal | 519 | 79.8 |
| In labor CS | 68 | 10.5 |
| Scheduled CS | 63 | 9.7 |

## 2.2 - Airway microbiome

### 2.2.1 - Subset airway samples

**air\_taxglm.RData** contains phyloseq objects with airway samples at phylum, genus and ASV level for both read counts and relative abundances, as well as a rarefied (2000 reads/sample) at ASV level.

### 2.2.2 - Dominant airway taxa and overall richness

#### 2.2.2.1 - tables

The distribution of the airway reads are here summarized on phylum, genus and individual ASV level.

Abundance of phyla, genera, and ASV in airway samples

| Included | Phylum | Genus | ASV |
| --- | --- | --- | --- |
| All | 35 | 828 | 7500 |
| > 0.01% | 13 | 107 | 288 |
| > 0.1% | 8 | 37 | 58 |
| > 1% | 4 | 10 | 13 |

Average abundance according to phylum

| Kingdom | Phylum | Abundance (%) |
| --- | --- | --- |
| Bacteria | Firmicutes | 60.8 |
| Bacteria | Proteobacteria | 30.4 |
| Bacteria | Actinobacteria | 5.5 |
| Bacteria | Bacteroidetes | 1.7 |
| Bacteria | Fusobacteria | 0.7 |
| Bacteria | Cyanobacteria | 0.3 |

Average abundance according to genus

| Kingdom | Phylum | Class | Order | Family | Genus | Abundance (%) |
| --- | --- | --- | --- | --- | --- | --- |
| Bacteria | Firmicutes | Bacilli | Bacillales | Staphylococcaceae | Staphylococcus | 25.6 |
| Bacteria | Firmicutes | Bacilli | Lactobacillales | Streptococcaceae | Streptococcus | 25.6 |
| Bacteria | Proteobacteria | Gammaproteobacteria | Pseudomonadales | Moraxellaceae | Moraxella | 14.8 |
| Bacteria | Proteobacteria | Gammaproteobacteria | Pasteurellales | Pasteurellaceae | Haemophilus | 6.0 |
| Bacteria | Actinobacteria | Actinobacteria | Corynebacteriales | Corynebacteriaceae | Corynebacterium | 4.1 |
| Bacteria | Firmicutes | Bacilli | Bacillales | Bacillales\_family\_XI | Gemella | 3.2 |

Average abundance according to ASV

| Kingdom | Phylum | Class | Order | Family | Genus | Species | ASV | Abundance (%) |
| --- | --- | --- | --- | --- | --- | --- | --- | --- |
| Bacteria | Firmicutes | Bacilli | Bacillales | Staphylococcaceae | Staphylococcus | Genus\_Staphylococcus | Genus\_Staphylococcus\_205 | 24.2 |
| Bacteria | Firmicutes | Bacilli | Lactobacillales | Streptococcaceae | Streptococcus | Genus\_Streptococcus | Genus\_Streptococcus\_177 | 18.4 |
| Bacteria | Proteobacteria | Gammaproteobacteria | Pseudomonadales | Moraxellaceae | Moraxella | Genus\_Moraxella | Genus\_Moraxella\_95 | 13.7 |
| Bacteria | Proteobacteria | Gammaproteobacteria | Pasteurellales | Pasteurellaceae | Haemophilus | Genus\_Haemophilus | Genus\_Haemophilus\_69 | 3.5 |
| Bacteria | Firmicutes | Bacilli | Lactobacillales | Streptococcaceae | Streptococcus | Streptococcus\_pneumoniae | Streptococcus\_pneumoniae\_98 | 3.4 |
| Bacteria | Firmicutes | Bacilli | Bacillales | Bacillales\_family\_XI | Gemella | Genus\_Gemella | Genus\_Gemella\_53 | 3.1 |

#### 2.2.2.2 - Plot

Figure 2A: Boxplot of top genera with mean abundance of 5% at least one time point. Abundance is plotted on a log10 scale, and all abundances below 0.1% have been set to 0.1% in the plot.

### 2.2.3 - Airway alpha diversity

For this part we use the rarefied samples (2000 reads/sample)

Summary of alpha diversity by time

|  |  | Observed Richness | | Shannon Diversity index | |
| --- | --- | --- | --- | --- | --- |
| Time | Samples (n) | Mean | SD | Mean | SD |
| One\_week | 526 | 16.580 | 9.532 | 1.114 | 0.635 |
| One\_month | 606 | 20.467 | 11.292 | 1.326 | 0.612 |
| Three\_months | 614 | 25.125 | 11.103 | 1.518 | 0.630 |

Summary of alpha diversity by time and delivery

|  |  | Observed Richness | | Shannon Diversity index | |
| --- | --- | --- | --- | --- | --- |
| Delivery mode | Samples (n) | Mean | SD | Mean | SD |
| **One\_week** | | | | | |
| In labor CS | 50 | 15.740 | 5.830 | 1.002 | 0.644 |
| Scheduled CS | 52 | 15.462 | 6.314 | 1.110 | 0.668 |
| Vaginal | 424 | 16.816 | 10.185 | 1.128 | 0.629 |
| **One\_month** | | | | | |
| In labor CS | 64 | 20.703 | 8.883 | 1.446 | 0.698 |
| Scheduled CS | 62 | 17.855 | 7.149 | 1.289 | 0.568 |
| Vaginal | 480 | 20.773 | 11.966 | 1.315 | 0.605 |
| **Three\_months** | | | | | |
| In labor CS | 65 | 25.677 | 11.741 | 1.560 | 0.663 |
| Scheduled CS | 60 | 22.750 | 10.388 | 1.462 | 0.653 |
| Vaginal | 489 | 25.344 | 11.088 | 1.519 | 0.624 |

Outliers not plotted in Figure 2B (Observed richness > 100)

| Child | Time point | Delivery | Mother’s CST (week 36) | Observed richness |
| --- | --- | --- | --- | --- |
| dyad464 | One\_month | Vaginal | CST\_I | 126 |
| dyad342 | One\_month | Vaginal | CST\_I | 153 |
| dyad408 | One\_week | Vaginal | CST\_III | 145 |

Figure 2 B-C: Boxplot of alpha diversity, (B) observed richness, and (C) Shannon diversity index, by delivery mode.

**Anova statistical test of alpha diversity by Time and Delivery**  
Overall differences in observed richness

```
## Analysis of Variance Table
## 
## Response: Observed
##             Df Sum Sq Mean Sq F value  Pr(>F)    
## Time         2  20891 10445.5 91.1252 < 2e-16 ***
## DELIVERY     2    846   423.0  3.6903 0.02516 *  
## Residuals 1741 199568   114.6                    
## ---
## Signif. codes:  0 '***' 0.001 '**' 0.01 '*' 0.05 '.' 0.1 ' ' 1
```

Multiple comparison of differences in observed richness

```
##   Tukey multiple comparisons of means
##     95% family-wise confidence level
## 
## Fit: aov(formula = Observed ~ Time + DELIVERY, data = df.adiv)
## 
## $Time
##                            diff      lwr       upr p adj
## One_month-One_week     3.887149 2.390517  5.383780     0
## Three_months-One_week  8.545559 7.053465 10.037653     0
## Three_months-One_month 4.658410 3.220340  6.096481     0
## 
## $DELIVERY
##                                diff        lwr       upr     p adj
## Scheduled CS-In labor CS -2.1341848 -4.8078501 0.5394805 0.1470299
## Vaginal-In labor CS       0.2037826 -1.7903087 2.1978739 0.9688195
## Vaginal-Scheduled CS      2.3379674  0.3186474 4.3572874 0.0183126
```

Overall differences in shannon diversity index

```
## Analysis of Variance Table
## 
## Response: Shannon
##             Df Sum Sq Mean Sq F value Pr(>F)    
## Time         2  46.25 23.1232 59.1001 <2e-16 ***
## DELIVERY     2   0.33  0.1652  0.4222 0.6557    
## Residuals 1741 681.18  0.3913                   
## ---
## Signif. codes:  0 '***' 0.001 '**' 0.01 '*' 0.05 '.' 0.1 ' ' 1
```

Multiple comparison of differences in shannon diversity index

```
##   Tukey multiple comparisons of means
##     95% family-wise confidence level
## 
## Fit: aov(formula = Shannon ~ Time + DELIVERY, data = df.adiv)
## 
## $Time
##                             diff       lwr       upr p adj
## One_month-One_week     0.2118842 0.1244465 0.2993219 0e+00
## Three_months-One_week  0.4039750 0.3168024 0.4911476 0e+00
## Three_months-One_month 0.1920908 0.1080744 0.2761072 3e-07
## 
## $DELIVERY
##                                 diff         lwr        upr     p adj
## Scheduled CS-In labor CS -0.06077442 -0.21697796 0.09542912 0.6323593
## Vaginal-In labor CS      -0.02598445 -0.14248524 0.09051634 0.8600079
## Vaginal-Scheduled CS      0.03478997 -0.08318476 0.15276469 0.7683547
```

Alpha diversity does not differ dependent on mothers vaginal CST at week 36

```
## Analysis of Variance Table
## 
## Response: Observed
##             Df Sum Sq Mean Sq F value Pr(>F)    
## Time         2  20891 10445.5 90.9177 <2e-16 ***
## CST_w36      5    735   147.0  1.2799 0.2698    
## Residuals 1738 199679   114.9                   
## ---
## Signif. codes:  0 '***' 0.001 '**' 0.01 '*' 0.05 '.' 0.1 ' ' 1
```

```
## Analysis of Variance Table
## 
## Response: Shannon
##             Df Sum Sq Mean Sq F value Pr(>F)    
## Time         2  46.25 23.1232 59.1229 <2e-16 ***
## CST_w36      5   1.77  0.3533  0.9033 0.4779    
## Residuals 1738 679.74  0.3911                   
## ---
## Signif. codes:  0 '***' 0.001 '**' 0.01 '*' 0.05 '.' 0.1 ' ' 1
```

### 2.2.4 - Airway beta diversity

#### 2.2.4.1 - Preparation

Calculation of Weighted UniFrac distances and NMDS ordination for airway samples

**air\_OrdinationRes.RData** contains weighted UniFrac distances and NMDS ordination of the airway samples

#### 2.2.4.2 - Plots and statistics

Figure 2D-E: Non-metric multidimensional scaling plots based on weighted UniFrac distances. Samples from the same individual are connected by gray lines. All plots are colored by time point.

PERMANOVA of Time using weighted Unifrac distances

```
## 
## Call:
## adonis(formula = air.WUnifrac ~ Time, data = df.plot, strata = df.plot$dyadnb) 
## 
## Blocks:  strata 
## Permutation: free
## Number of permutations: 999
## 
## Terms added sequentially (first to last)
## 
##             Df SumsOfSqs   MeanSqs F.Model      R2 Pr(>F)    
## Time         2   0.01549 0.0077448  11.701 0.01325  0.001 ***
## Residuals 1743   1.15367 0.0006619         0.98675           
## Total     1745   1.16916                   1.00000           
## ---
## Signif. codes:  0 '***' 0.001 '**' 0.01 '*' 0.05 '.' 0.1 ' ' 1
```

PERMANOVA of mothers CST at week 36 using weighted Unifrac distances

```
## 
## Call:
## adonis(formula = air.WUnifrac ~ CST_w36, data = df.plot, strata = df.plot$dyadnb) 
## 
## Blocks:  strata 
## Permutation: free
## Number of permutations: 999
## 
## Terms added sequentially (first to last)
## 
##             Df SumsOfSqs    MeanSqs F.Model      R2 Pr(>F)
## CST_w36      5   0.00465 0.00093031  1.3901 0.00398      1
## Residuals 1740   1.16451 0.00066926         0.99602       
## Total     1745   1.16916                    1.00000
```

```
## 
## Call:
## adonis(formula = air.WUnifrac ~ Time * CST_w36, data = df.plot,      strata = df.plot$dyadnb) 
## 
## Blocks:  strata 
## Permutation: free
## Number of permutations: 999
## 
## Terms added sequentially (first to last)
## 
##                Df SumsOfSqs   MeanSqs F.Model      R2 Pr(>F)    
## Time            2   0.01549 0.0077448 11.6933 0.01325  0.001 ***
## CST_w36         5   0.00454 0.0009089  1.3722 0.00389  0.926    
## Time:CST_w36   10   0.00463 0.0004630  0.6990 0.00396  0.862    
## Residuals    1728   1.14450 0.0006623         0.97890           
## Total        1745   1.16916                   1.00000           
## ---
## Signif. codes:  0 '***' 0.001 '**' 0.01 '*' 0.05 '.' 0.1 ' ' 1
```

PERMANOVA of Delivery using weighted Unifrac distances

```
## 
## Call:
## adonis(formula = air.WUnifrac ~ DELIVERY, data = df.plot, strata = df.plot$dyadnb) 
## 
## Blocks:  strata 
## Permutation: free
## Number of permutations: 999
## 
## Terms added sequentially (first to last)
## 
##             Df SumsOfSqs    MeanSqs F.Model      R2 Pr(>F)
## DELIVERY     2   0.00287 0.00143643  2.1467 0.00246      1
## Residuals 1743   1.16629 0.00066913         0.99754       
## Total     1745   1.16916                    1.00000
```

```
## 
## Call:
## adonis(formula = air.WUnifrac ~ Time * DELIVERY, data = df.plot,      strata = df.plot$dyadnb) 
## 
## Blocks:  strata 
## Permutation: free
## Number of permutations: 999
## 
## Terms added sequentially (first to last)
## 
##                 Df SumsOfSqs   MeanSqs F.Model      R2 Pr(>F)    
## Time             2   0.01549 0.0077448 11.7102 0.01325  0.001 ***
## DELIVERY         2   0.00278 0.0013891  2.1004 0.00238  0.960    
## Time:DELIVERY    4   0.00210 0.0005242  0.7926 0.00179  0.444    
## Residuals     1737   1.14879 0.0006614         0.98258           
## Total         1745   1.16916                   1.00000           
## ---
## Signif. codes:  0 '***' 0.001 '**' 0.01 '*' 0.05 '.' 0.1 ' ' 1
```

### 2.2.5 - Create figure 2 illustrating airway microbiomes

Figure 2: Airway microbiome. (A) Boxplot of top genera with mean abundance of 5% at least one time point. Abundance is plotted on a log10 scale, and all abundances below 0.1% have been set to 0.1% in the plot. (B–C) Boxplot of alpha diversity, (B) observed richness, and (C) Shannon diversity index, by delivery mode. Three samples with observed richness above 100 were excluded from the plot. (D–E) Non-metric multidimensional scaling plots based on weighted UniFrac distances. Samples from the same individual are connected by gray lines. All plots are colored by time point.

```
## png 
##   2
```

## 2.3 - Fecal microbiome

### 2.3.1 - Subset fecal samples

**fec\_taxglm.RData** contains phyloseq objects with fecal samples at phylum, genus and ASV level for both read counts and relative abundances, as well as a rarefied (2000 reads/sample) at ASV level.

### 2.3.2 - Dominant fecal taxa and overall richness

#### 2.3.2.1 - tables

The distribution of the fecal reads are here summarized on phylum, genus and individual ASV level.

Count of phyla, genera, and ASV in fecal samples

| Included | Phylum | Genus | ASV |
| --- | --- | --- | --- |
| All | 33 | 707 | 6818 |
| > 0.01% | 8 | 105 | 306 |
| > 0.1% | 5 | 44 | 87 |
| > 1% | 5 | 14 | 22 |

Average abundance according to phylum

| Kingdom | Phylum | Abundance (%) |
| --- | --- | --- |
| Bacteria | Bacteroidetes | 34.4 |
| Bacteria | Proteobacteria | 26.4 |
| Bacteria | Firmicutes | 21.4 |
| Bacteria | Actinobacteria | 16.2 |
| Bacteria | Verrucomicrobia | 1.4 |
| Bacteria | Fusobacteria | 0.1 |

Average abundance according to genus

| Kingdom | Phylum | Class | Order | Family | Genus | Abundance (%) |
| --- | --- | --- | --- | --- | --- | --- |
| Bacteria | Bacteroidetes | Bacteroidia | Bacteroidales | Bacteroidaceae | Bacteroides | 29.2 |
| Bacteria | Actinobacteria | Actinobacteria | Bifidobacteriales | Bifidobacteriaceae | Bifidobacterium | 15.6 |
| Bacteria | Proteobacteria | Gammaproteobacteria | Enterobacteriales | Enterobacteriaceae | Escherichia\_Shigella | 14.1 |
| Bacteria | Proteobacteria | Gammaproteobacteria | Enterobacteriales | Enterobacteriaceae | Family\_Enterobacteriaceae | 9.2 |
| Bacteria | Firmicutes | Negativicutes | Selenomonadales | Veillonellaceae | Veillonella | 3.3 |
| Bacteria | Firmicutes | Clostridia | Clostridiales | Clostridiaceae\_1 | Clostridium\_ss\_1 | 3.0 |

Average abundance according to ASV

| Kingdom | Phylum | Class | Order | Family | Genus | Species | ASV | Abundance (%) |
| --- | --- | --- | --- | --- | --- | --- | --- | --- |
| Bacteria | Proteobacteria | Gammaproteobacteria | Enterobacteriales | Enterobacteriaceae | Escherichia\_Shigella | Genus\_Escherichia\_Shigella | Genus\_Escherichia\_Shigella\_101 | 13.2 |
| Bacteria | Actinobacteria | Actinobacteria | Bifidobacteriales | Bifidobacteriaceae | Bifidobacterium | Genus\_Bifidobacterium | Genus\_Bifidobacterium\_60 | 10.0 |
| Bacteria | Bacteroidetes | Bacteroidia | Bacteroidales | Bacteroidaceae | Bacteroides | Bacteroides\_fragilis | Bacteroides\_fragilis\_22 | 6.6 |
| Bacteria | Bacteroidetes | Bacteroidia | Bacteroidales | Bacteroidaceae | Bacteroides | Genus\_Bacteroides | Genus\_Bacteroides\_293 | 6.3 |
| Bacteria | Bacteroidetes | Bacteroidia | Bacteroidales | Bacteroidaceae | Bacteroides | Genus\_Bacteroides | Genus\_Bacteroides\_260 | 5.1 |
| Bacteria | Proteobacteria | Gammaproteobacteria | Enterobacteriales | Enterobacteriaceae | Family\_Enterobacteriaceae | Family\_Enterobacteriaceae | Family\_Enterobacteriaceae\_63 | 4.6 |

#### 2.3.2.2 - Plot

Figure 3A: Fecal sample composition. Boxplot of top genera with mean abundance of 4% at least one time point. Abundance is plotted on a log10 scale, and all abundances below 0.1% have been set to 0.1% in the plot.

### 2.3.3 - Fecal alpha diversity

For this part we use the rarefied samples (2000 reads/sample)

Summary of alpha diversity by time

|  |  | Observed Richness | | Shannon Diversity index | |
| --- | --- | --- | --- | --- | --- |
| Time | Samples (n) | Mean | SD | Mean | SD |
| One\_week | 533 | 23.206 | 14.263 | 1.515 | 0.614 |
| One\_month | 575 | 22.101 | 9.367 | 1.400 | 0.541 |
| One\_year | 580 | 53.150 | 18.538 | 2.327 | 0.597 |

Summary of alpha diversity by time and delivery

|  |  | Observed Richness | | Shannon Diversity index | |
| --- | --- | --- | --- | --- | --- |
| Delivery mode | Samples (n) | Mean | SD | Mean | SD |
| **One\_week** | | | | | |
| In labor CS | 56 | 23.482 | 11.557 | 1.473 | 0.658 |
| Scheduled CS | 51 | 25.725 | 32.563 | 1.563 | 0.773 |
| Vaginal | 426 | 22.869 | 10.570 | 1.515 | 0.588 |
| **One\_month** | | | | | |
| In labor CS | 64 | 21.672 | 8.142 | 1.301 | 0.470 |
| Scheduled CS | 50 | 21.540 | 9.008 | 1.399 | 0.596 |
| Vaginal | 461 | 22.221 | 9.576 | 1.414 | 0.543 |
| **One\_year** | | | | | |
| In labor CS | 60 | 49.883 | 18.762 | 2.271 | 0.666 |
| Scheduled CS | 54 | 53.130 | 18.763 | 2.329 | 0.611 |
| Vaginal | 466 | 53.573 | 18.482 | 2.335 | 0.587 |

Outlier not plotted in Figure 3B (Observed richness > 150)

| Child | Time point | Delivery | Mother’s CST (week 36) | Observed richness |
| --- | --- | --- | --- | --- |
| dyad734 | One\_week | Scheduled CS | CST\_II | 246 |

Figure 3 B-C: Boxplot of alpha diversity, (B) observed richness, and (C) Shannon diversity index, by delivery mode. One sample with observed richness of >150 was excluded from the plot.

**Anova statistical test of alpha diversity by Time and Delivery**  
Overall differences in observed richness

```
## [1] "Statistical test of alpha diversity by Time and Delivery method"
```

```
## Analysis of Variance Table
## 
## Response: Observed
##             Df Sum Sq Mean Sq  F value Pr(>F)    
## Time         2 354897  177448 835.9279 <2e-16 ***
## DELIVERY     2    313     156   0.7369 0.4788    
## Residuals 1683 357263     212                    
## ---
## Signif. codes:  0 '***' 0.001 '**' 0.01 '*' 0.05 '.' 0.1 ' ' 1
```

Multiple comparison of differences in observed richness

```
##   Tukey multiple comparisons of means
##     95% family-wise confidence level
## 
## Fit: aov(formula = Observed ~ Time + DELIVERY, data = df.adiv)
## 
## $Time
##                         diff       lwr        upr     p adj
## One_month-One_week -1.105509 -3.160507  0.9494882 0.4170142
## One_year-One_week  29.943621 27.892889 31.9943532 0.0000000
## One_year-One_month 31.049130 29.037806 33.0604545 0.0000000
## 
## $DELIVERY
##                               diff       lwr      upr     p adj
## Scheduled CS-In labor CS  1.796473 -1.948591 5.541538 0.4985965
## Vaginal-In labor CS       1.235954 -1.475642 3.947549 0.5333632
## Vaginal-Scheduled CS     -0.560520 -3.458697 2.337657 0.8927733
```

Overall differences in shannon diversity index

```
## Analysis of Variance Table
## 
## Response: Shannon
##             Df Sum Sq Mean Sq  F value Pr(>F)    
## Time         2 293.27 146.636 429.8028 <2e-16 ***
## DELIVERY     2   0.92   0.462   1.3549 0.2583    
## Residuals 1683 574.19   0.341                    
## ---
## Signif. codes:  0 '***' 0.001 '**' 0.01 '*' 0.05 '.' 0.1 ' ' 1
```

Multiple comparison of differences in shannon diversity index

```
##   Tukey multiple comparisons of means
##     95% family-wise confidence level
## 
## Fit: aov(formula = Shannon ~ Time + DELIVERY, data = df.adiv)
## 
## $Time
##                          diff        lwr         upr     p adj
## One_month-One_week -0.1149787 -0.1973632 -0.03259411 0.0031055
## One_year-One_week   0.8125265  0.7303130  0.89474009 0.0000000
## One_year-One_month  0.9275052  0.8468715  1.00813890 0.0000000
## 
## $DELIVERY
##                                  diff         lwr       upr     p adj
## Scheduled CS-In labor CS  0.083511039 -0.06662806 0.2336501 0.3926698
## Vaginal-In labor CS       0.074407660 -0.03429982 0.1831151 0.2434904
## Vaginal-Scheduled CS     -0.009103379 -0.12529088 0.1070841 0.9815499
```

Alpha diversity does not differ dependent on mothers vaginal CST at week 36

```
## Analysis of Variance Table
## 
## Response: Observed
##             Df Sum Sq Mean Sq  F value Pr(>F)    
## Time         2 354897  177448 836.2606 <2e-16 ***
## CST_w36      5   1092     218   1.0288 0.3989    
## Residuals 1680 356484     212                    
## ---
## Signif. codes:  0 '***' 0.001 '**' 0.01 '*' 0.05 '.' 0.1 ' ' 1
```

```
## Analysis of Variance Table
## 
## Response: Shannon
##             Df Sum Sq Mean Sq  F value Pr(>F)    
## Time         2 293.27 146.636 429.0059 <2e-16 ***
## CST_w36      5   0.88   0.177   0.5169 0.7637    
## Residuals 1680 574.23   0.342                    
## ---
## Signif. codes:  0 '***' 0.001 '**' 0.01 '*' 0.05 '.' 0.1 ' ' 1
```

### 2.3.4 - Fecal beta diversity

#### 2.3.4.1 - Preparation

Calculation of Weighted UniFrac distances and NMDS ordination for fecal samples

**fec\_OrdinationRes.RData** contains weighted UniFrac distances and NMDS ordination of the fecal samples

#### 2.3.4.2 - Plots and statistics

Figure 3D-E: Non-metric multi-dimensional scaling plot based on weighted UniFrac distances. Samples from the same individual are connected by gray lines.

PERMANOVA of Time using weighted Unifrac distances

```
## 
## Call:
## adonis(formula = fec.WUnifrac ~ Time, data = df.plot, strata = df.plot$dyadnb) 
## 
## Blocks:  strata 
## Permutation: free
## Number of permutations: 999
## 
## Terms added sequentially (first to last)
## 
##             Df SumsOfSqs   MeanSqs F.Model      R2 Pr(>F)    
## Time         2   0.05639 0.0281953  30.617 0.03507  0.001 ***
## Residuals 1685   1.55172 0.0009209         0.96493           
## Total     1687   1.60811                   1.00000           
## ---
## Signif. codes:  0 '***' 0.001 '**' 0.01 '*' 0.05 '.' 0.1 ' ' 1
```

PERMANOVA of mothers CST at week 36 using weighted Unifrac distances

```
## 
## Call:
## adonis(formula = fec.WUnifrac ~ CST_w36, data = df.plot, strata = df.plot$dyadnb) 
## 
## Blocks:  strata 
## Permutation: free
## Number of permutations: 999
## 
## Terms added sequentially (first to last)
## 
##             Df SumsOfSqs    MeanSqs F.Model      R2 Pr(>F)
## CST_w36      5   0.01092 0.00218339  2.2993 0.00679      1
## Residuals 1682   1.59720 0.00094958         0.99321       
## Total     1687   1.60811                    1.00000
```

```
## 
## Call:
## adonis(formula = fec.WUnifrac ~ Time * CST_w36, data = df.plot,      strata = df.plot$dyadnb) 
## 
## Blocks:  strata 
## Permutation: free
## Number of permutations: 999
## 
## Terms added sequentially (first to last)
## 
##                Df SumsOfSqs   MeanSqs F.Model      R2 Pr(>F)    
## Time            2   0.05639 0.0281953 30.7926 0.03507  0.001 ***
## CST_w36         5   0.01162 0.0023244  2.5386 0.00723  0.001 ***
## Time:CST_w36   10   0.01096 0.0010964  1.1974 0.00682  0.546    
## Residuals    1670   1.52914 0.0009157         0.95089           
## Total        1687   1.60811                   1.00000           
## ---
## Signif. codes:  0 '***' 0.001 '**' 0.01 '*' 0.05 '.' 0.1 ' ' 1
```

PERMANOVA of Delivery using weighted Unifrac distances

```
## 
## Call:
## adonis(formula = fec.WUnifrac ~ DELIVERY, data = df.plot, strata = df.plot$dyadnb) 
## 
## Blocks:  strata 
## Permutation: free
## Number of permutations: 999
## 
## Terms added sequentially (first to last)
## 
##             Df SumsOfSqs   MeanSqs F.Model      R2 Pr(>F)
## DELIVERY     2   0.00703 0.0035144  3.6986 0.00437      1
## Residuals 1685   1.60108 0.0009502         0.99563       
## Total     1687   1.60811                   1.00000
```

```
## 
## Call:
## adonis(formula = fec.WUnifrac ~ Time * DELIVERY, data = df.plot,      strata = df.plot$dyadnb) 
## 
## Blocks:  strata 
## Permutation: free
## Number of permutations: 999
## 
## Terms added sequentially (first to last)
## 
##                 Df SumsOfSqs   MeanSqs F.Model      R2 Pr(>F)    
## Time             2   0.05639 0.0281953 30.9488 0.03507  0.001 ***
## DELIVERY         2   0.00698 0.0034906  3.8315 0.00434  0.001 ***
## Time:DELIVERY    4   0.01512 0.0037808  4.1500 0.00940  0.001 ***
## Residuals     1679   1.52962 0.0009110         0.95119           
## Total         1687   1.60811                   1.00000           
## ---
## Signif. codes:  0 '***' 0.001 '**' 0.01 '*' 0.05 '.' 0.1 ' ' 1
```

### 2.3.5 - Create figure 3 illustrating fecal microbiomes

Figure 3: Fecal microbiome. (A) Boxplot of top genera with mean abundance of 4% at least one time point. Abundance is plotted on a log10 scale, and all abundances below 0.1% have been set to 0.1% in the plot. (B–C) Boxplot of alpha diversity, (B) observed richness, and (C) Shannon diversity index, by delivery mode. One sample with observed richness of >150 was excluded from the plot. (D–E) Non-metric multi-dimensional scaling plot based on weighted UniFrac distances. Samples from the same individual are connected by gray lines. All plots are colored by time point.

```
## png 
##   2
```

# 3 - Transfer

## 3.1 - Preparation

### 3.1.1 - Calculation of ALL individual ASV models

This is the foundation of all further analysis of transfer from mother to infant Output of this section in ‘./ORresults.RData’

**ORresults.RData** contains all calculated odds ratios for transfer across delivery mode, infant sample type and time point

### 3.1.2 - Permutational inference calculation

A permutation test between c-sectio and vaginal birth are conducted for all combinations.

#### 3.1.2.1 - Calculations

**weighted\_permutation\_results\_onesided.RData** contains the permutations results for the transfer odds

#### 3.1.2.2 - Format output

**STATtot.RData** contains the formated transfer results as well as supporting tables and lists

#### 3.1.2.3 - Overview of testable ASVs

Table 1 - testable ASV’s

Table1: Individual transfer models, coverage of testable ASVs and strongest results

|  |  |  | Relative abundance\* | | Minimum p-value† | | ASVs with p-value below cutoff† | | | |
| --- | --- | --- | --- | --- | --- | --- | --- | --- | --- | --- |
| Time | Type | ASVs(n) | Mother | Child | crude | adjusted | crude < 0.01 | crude < 0.05 | adjusted < 0.05 | adjusted < 0.1 |
| **Cesarean sectio** | | | | | | | | | | |
| 7 | Airways | 104 | 30.994 | 36.451 | 0.058 | 0.990 | 0 | 0 | 0 | 0 |
| 7 | Fecal | 160 | 31.606 | 73.259 | 0.000 | 0.008 | 3 | 5 | 1 | 1 |
| 30 | Airways | 131 | 56.383 | 84.976 | 0.008 | 0.347 | 3 | 6 | 0 | 0 |
| 30 | Fecal | 181 | 60.471 | 83.228 | 0.008 | 0.991 | 1 | 4 | 0 | 0 |
| 90 | Airways | 152 | 56.791 | 84.419 | 0.033 | 0.992 | 0 | 3 | 0 | 0 |
| 300 | Fecal | 161 | 61.669 | 59.292 | 0.018 | 0.991 | 0 | 2 | 0 | 0 |
| **Vaginal delivery** | | | | | | | | | | |
| 7 | Airways | 293 | 63.970 | 45.965 | 0.002 | 0.691 | 3 | 13 | 0 | 0 |
| 7 | Fecal | 354 | 65.154 | 90.692 | 0.000 | 0.012 | 12 | 28 | 2 | 4 |
| 30 | Airways | 342 | 63.859 | 90.155 | 0.000 | 0.001 | 8 | 14 | 1 | 2 |
| 30 | Fecal | 395 | 65.809 | 92.392 | 0.002 | 0.312 | 11 | 28 | 0 | 0 |
| 90 | Airways | 364 | 62.224 | 87.668 | 0.001 | 0.260 | 3 | 9 | 0 | 0 |
| 300 | Fecal | 404 | 64.182 | 84.451 | 0.003 | 0.457 | 7 | 17 | 0 | 0 |
|  |
| --- |
| \* Mean relative abundance of the testable ASVs in mother or child |
| † Crude p-values are the direct output of each transfer model, adjusted p-values have been FDR adjusted |

Descriptives on testable Amplicon sequence variant (ASV) in terms of numbers of ASVs, vaginal, fecal, and airway total coverage, number of tests reaching nominal, and false discovery rate-corrected significance.

|  |  |  | Relative abundance\* | | Minimum p-value† | | ASVs with p-value below cutoff† | | | |
| --- | --- | --- | --- | --- | --- | --- | --- | --- | --- | --- |
| Time | Type | ASVs(n) | Mother | Child | crude | adjusted | crude < 0.01 | crude < 0.05 | adjusted < 0.05 | adjusted < 0.1 |
| **In labor cesarean sectio** | | | | | | | | | | |
| 7 | Airways | 65 | 20.616 | 16.849 | 0.171 | 0.980 | 0 | 0 | 0 | 0 |
| 7 | Fecal | 105 | 21.214 | 63.576 | 0.001 | 0.068 | 1 | 2 | 0 | 1 |
| 30 | Airways | 81 | 50.822 | 59.373 | 0.005 | 0.374 | 1 | 4 | 0 | 0 |
| 30 | Fecal | 121 | 55.910 | 75.822 | 0.016 | 0.984 | 0 | 3 | 0 | 0 |
| 90 | Airways | 105 | 50.661 | 85.466 | 0.052 | 0.985 | 0 | 0 | 0 | 0 |
| 300 | Fecal | 100 | 58.004 | 50.603 | 0.017 | 0.983 | 0 | 4 | 0 | 0 |
| **Scheduled cesarean sectio** | | | | | | | | | | |
| 7 | Airways | 58 | 30.109 | 36.183 | 0.038 | 0.981 | 0 | 1 | 0 | 0 |
| 7 | Fecal | 89 | 28.119 | 58.184 | 0.020 | 0.873 | 0 | 3 | 0 | 0 |
| 30 | Airways | 75 | 23.747 | 81.034 | 0.016 | 0.984 | 0 | 2 | 0 | 0 |
| 30 | Fecal | 107 | 30.836 | 46.935 | 0.037 | 0.980 | 0 | 1 | 0 | 0 |
| 90 | Airways | 87 | 28.459 | 76.014 | 0.033 | 0.983 | 0 | 1 | 0 | 0 |
| 300 | Fecal | 91 | 35.645 | 33.860 | 0.054 | 0.981 | 0 | 0 | 0 | 0 |
|  |
| --- |
| \* Mean relative abundance of the testable ASVs in mother or child |
| † Crude p-values are the direct output of each transfer model, adjusted p-values have been FDR adjusted |

#### 3.1.2.4 - ASVs with significant transfer odds

Transfer models that were significant after FDR correction

|  |  |  | Counts\* | | | | Taxonomy | | | | | | |
| --- | --- | --- | --- | --- | --- | --- | --- | --- | --- | --- | --- | --- | --- |
| Time | Type | Delivery | 0/0 | 1/0 | 0/1 | 1/1 | Phylum | Class | Order | Family | Genus | Species | ASV |
| 7 | Fecal | CS | 102 | 2 | 0 | 3 | Proteobacteria | Gammaproteobacteria | Enterobacteriales | Enterobacteriaceae | Escherichia\_Shigella | Genus\_Escherichia\_Shigella | Genus\_Escherichia\_Shigella\_145 |
| 7 | Fecal | Vaginal | 423 | 1 | 0 | 2 | Proteobacteria | Gammaproteobacteria | Cardiobacteriales | Wohlfahrtiimonadaceae | Koukoulia | Genus\_Koukoulia | Genus\_Koukoulia\_3 |
| 7 | Fecal | Vaginal | 265 | 144 | 3 | 14 | Bacteroidetes | Bacteroidia | Bacteroidales | Prevotellaceae | Prevotella | Genus\_Prevotella | Genus\_Prevotella\_20 |
| 30 | Airways | Vaginal | 284 | 149 | 14 | 33 | Tenericutes | Mollicutes | Mycoplasmatales | Mycoplasmataceae | Ureaplasma | Genus\_Ureaplasma | Genus\_Ureaplasma\_8 |
|  |
| --- |
| \* Count of pairs in which the ASV is not found (0/0), found only in the mother (1/0), only in the child (0/1), or in both mother and child (1/1) |

All transfer models for above mentioned ASVs

|  |  |  | Counts\* | | | | P-values† | |
| --- | --- | --- | --- | --- | --- | --- | --- | --- |
| Time | Type | Delivery | 0/0 | 1/0 | 0/1 | 1/1 | crude | adjusted |
| **Genus\_Escherichia\_Shigella\_145** | | | | | | | | |
| 7 | Airways | CS | 96 | 5 | 1 | 0 | 0.95098 | 0.99020 |
| 7 | Fecal | CS | 102 | 2 | 0 | 3 | 0.00005 | 0.00806 |
| 30 | Airways | CS | 121 | 4 | 0 | 1 | 0.03968 | 0.86640 |
| 30 | Fecal | CS | 104 | 3 | 5 | 2 | 0.02977 | 0.99123 |
| 90 | Airways | CS | 120 | 4 | 1 | 0 | 0.96800 | 0.99200 |
| 300 | Fecal | CS | 107 | 3 | 3 | 1 | 0.13483 | 0.99123 |
| 7 | Airways | In labor CS | 47 | 2 | 1 | 0 | 0.96000 | 0.98000 |
| 7 | Fecal | In labor CS | 54 | 0 | 0 | 2 | 0.00065 | 0.06818 |
| 30 | Airways | In labor CS | 62 | 1 | 0 | 1 | 0.03125 | 0.84375 |
| 30 | Fecal | In labor CS | 61 | 1 | 1 | 1 | 0.06200 | 0.98437 |
| 300 | Fecal | In labor CS | 57 | 1 | 1 | 1 | 0.06610 | 0.98333 |
| 7 | Fecal | Scheduled CS | 48 | 2 | 0 | 1 | 0.05882 | 0.98039 |
| 30 | Fecal | Scheduled CS | 43 | 2 | 4 | 1 | 0.27602 | 0.98000 |
| 90 | Airways | Scheduled CS | 56 | 3 | 1 | 0 | 0.95000 | 0.98333 |
| 300 | Fecal | Scheduled CS | 50 | 2 | 2 | 0 | 0.92662 | 0.98148 |
| 7 | Airways | Vaginal | 409 | 14 | 0 | 1 | 0.03538 | 0.79735 |
| 7 | Fecal | Vaginal | 397 | 18 | 10 | 1 | 0.39831 | 0.99765 |
| 30 | Fecal | Vaginal | 427 | 18 | 14 | 2 | 0.14909 | 0.99783 |
| 300 | Fecal | Vaginal | 443 | 21 | 2 | 0 | 0.91181 | 0.99785 |
| **Genus\_Koukoulia\_3** | | | | | | | | |
| 7 | Fecal | CS | 105 | 1 | 1 | 0 | 0.99065 | 0.99065 |
| 30 | Airways | CS | 123 | 1 | 2 | 0 | 0.98413 | 0.99206 |
| 30 | Airways | Scheduled CS | 60 | 1 | 1 | 0 | 0.98387 | 0.98387 |
| 7 | Fecal | Vaginal | 423 | 1 | 0 | 2 | 0.00003 | 0.01173 |
| 30 | Airways | Vaginal | 473 | 3 | 4 | 0 | 0.97516 | 0.99792 |
| **Genus\_Prevotella\_20** | | | | | | | | |
| 7 | Fecal | CS | 58 | 47 | 1 | 1 | 0.69829 | 0.99065 |
| 30 | Fecal | CS | 62 | 46 | 3 | 3 | 0.51872 | 0.99123 |
| 90 | Airways | CS | 68 | 54 | 2 | 1 | 0.59029 | 0.99200 |
| 300 | Fecal | CS | 58 | 50 | 4 | 2 | 0.42658 | 0.99123 |
| 7 | Fecal | In labor CS | 31 | 23 | 1 | 1 | 0.67792 | 0.98214 |
| 30 | Fecal | In labor CS | 36 | 26 | 1 | 1 | 0.66964 | 0.98437 |
| 90 | Airways | In labor CS | 36 | 27 | 1 | 1 | 0.67981 | 0.98462 |
| 300 | Fecal | In labor CS | 31 | 25 | 3 | 1 | 0.41416 | 0.98333 |
| 30 | Fecal | Scheduled CS | 26 | 20 | 2 | 2 | 0.59815 | 0.98000 |
| 90 | Airways | Scheduled CS | 32 | 27 | 1 | 0 | 0.55000 | 0.98333 |
| 300 | Fecal | Scheduled CS | 27 | 25 | 1 | 1 | 0.73585 | 0.98148 |
| 7 | Airways | Vaginal | 268 | 155 | 1 | 0 | 0.63443 | 0.99764 |
| 7 | Fecal | Vaginal | 265 | 144 | 3 | 14 | 0.00013 | 0.02353 |
| 30 | Airways | Vaginal | 300 | 175 | 2 | 3 | 0.26749 | 0.99792 |
| 30 | Fecal | Vaginal | 268 | 151 | 27 | 15 | 0.55546 | 0.99783 |
| 90 | Airways | Vaginal | 313 | 172 | 2 | 2 | 0.44748 | 0.99796 |
| 300 | Fecal | Vaginal | 282 | 163 | 13 | 8 | 0.53032 | 0.99785 |
| **Genus\_Ureaplasma\_8** | | | | | | | | |
| 7 | Airways | CS | 65 | 35 | 0 | 2 | 0.12930 | 0.99020 |
| 30 | Airways | CS | 78 | 44 | 0 | 4 | 0.01944 | 0.63667 |
| 90 | Airways | CS | 79 | 42 | 1 | 3 | 0.13251 | 0.99200 |
| 7 | Airways | In labor CS | 28 | 20 | 0 | 2 | 0.18857 | 0.98000 |
| 30 | Airways | In labor CS | 34 | 27 | 0 | 3 | 0.09745 | 0.98437 |
| 90 | Airways | In labor CS | 34 | 28 | 1 | 2 | 0.44151 | 0.98462 |
| 30 | Airways | Scheduled CS | 44 | 17 | 0 | 1 | 0.29032 | 0.98387 |
| 90 | Airways | Scheduled CS | 45 | 14 | 0 | 1 | 0.25000 | 0.98333 |
| 7 | Airways | Vaginal | 269 | 154 | 1 | 0 | 0.63679 | 0.99764 |
| 30 | Airways | Vaginal | 284 | 149 | 14 | 33 | 0.00000 | 0.00081 |
| 30 | Fecal | Vaginal | 290 | 169 | 0 | 2 | 0.13708 | 0.99783 |
| 90 | Airways | Vaginal | 296 | 161 | 11 | 21 | 0.00071 | 0.25976 |
|  |
| --- |
| Note: |
| Transfer models which were significant after FDR are highlighted in bold |
| \* Count of pairs in which the ASV is not found (0/0), found only in the mother (1/0), only in the child (0/1), or in both mother and child (1/1) |
| † Crude p-values are the direct output of each transfer model, adjusted p-values have been FDR adjusted |

### 3.1.3 - Plot ASV transfer odds

The odds for transfer between mother (week 36) and child. Top panel shows the OR (x-axis) and the strength (p-value). Lower panel shows OR (y-axis) versus the population wide vaginal abundance (x-axis). This shows, that 1) there is trend of transfer from more ASVs being positive (OR>1) than negative, more signal in fecal, and that those which obtain the strongest transfer results are those which are in low population-wide vaginal abundance.

#### 3.1.3.1 - Figure 4-figure supplement 1 - vaginal delivery

Figure 4-figure supplement 1: The odds for transfer between mother (week 36) and child for children delivered vaginally. Top panel shows the odds ratio (OR) (x-axis) and the strength (p-value). Lower panel shows OR (y-axis) versus the population-wide vaginal abundance (x-axis).

#### 3.1.3.2 - Figure 4-figure supplement 2 - sectio delivery

Figure 4-figure supplement 2: The odds for transfer between mother (week 36) and child for children delivered by cesarean section (CS). Top panel shows the OR (x-axis) and the strength (p-value). Lower panel shows OR (y-axis) versus the population-wide vaginal abundance (x-axis).

#### 3.1.3.3 - Figure 4-figure supplement 3 - In labor sectio delivery

Figure 4-figure supplement 3: The odds for transfer between mother (week 36) and child for children delivered by in labor CS. Top panel shows the OR (x-axis) and the strength (p-value). Lower panel shows OR (y-axis) versus the population-wide vaginal abundance (x-axis).

#### 3.1.3.4 - Figure 4-figure supplement 4 - Scheduled sectio delivery

Figure 4-figure supplement 4: The odds for transfer between mother (week 36) and child for children delivered by scheduled CS. Top panel shows the OR (x-axis) and the strength (p-value). Lower panel shows OR (y-axis) versus the population-wide vaginal abundance (x-axis).

#### 3.1.3.5 - Figure 4-figure supplement statistics

Inference for relation between odds for tranfers and population maternal abundance\*

| Delivery | Time (days) | log10(abundance)† | SE‡ | p-value |
| --- | --- | --- | --- | --- |
| **Airways** | | | | |
| CS | 7 | 0.2101 | 0.1135 | 0.0669 |
| CS | 30 | 0.0193 | 0.0950 | 0.8391 |
| CS | 90 | -0.1157 | 0.0815 | 0.1580 |
| In labor CS | 7 | 0.4703 | 0.1387 | 0.0012 |
| In labor CS | 30 | -0.2729 | 0.1453 | 0.0640 |
| In labor CS | 90 | -0.1132 | 0.1123 | 0.3157 |
| Scheduled CS | 7 | -0.2807 | 0.1431 | 0.0549 |
| Scheduled CS | 30 | 0.1851 | 0.1256 | 0.1448 |
| Scheduled CS | 90 | 0.1479 | 0.1310 | 0.2620 |
| Vaginal | 7 | -0.0114 | 0.0559 | 0.8385 |
| Vaginal | 30 | 0.0686 | 0.0481 | 0.1543 |
| Vaginal | 90 | -0.0279 | 0.0553 | 0.6142 |
| **Fecal** | | | | |
| CS | 7 | 0.0120 | 0.0813 | 0.8832 |
| CS | 30 | -0.0535 | 0.0722 | 0.4595 |
| CS | 300 | -0.2048 | 0.0834 | 0.0151 |
| In labor CS | 7 | 0.1879 | 0.1319 | 0.1575 |
| In labor CS | 30 | -0.1311 | 0.1018 | 0.2003 |
| In labor CS | 300 | -0.1030 | 0.1296 | 0.4287 |
| Scheduled CS | 7 | -0.1708 | 0.1080 | 0.1174 |
| Scheduled CS | 30 | -0.0570 | 0.1189 | 0.6329 |
| Scheduled CS | 300 | -0.0015 | 0.1258 | 0.9903 |
| Vaginal | 7 | -0.1048 | 0.0525 | 0.0466 |
| Vaginal | 30 | -0.1862 | 0.0466 | 0.0001 |
| Vaginal | 300 | -0.1565 | 0.0513 | 0.0024 |
|  |
| --- |
| \* Linear models of maternal abundance (log10 transformed) and WTR, significant models are highlighted in bold |
| † Log10 transformed relative maternal abundance |
| ‡ Standard error |

## 3.2 - Weighted Odds Ratio

In order to make a commen measure for the tranfer signal, a weigthed transfer ratio (WTR) for each compartment and delivery mode. WTR were defined as WP/WN, where WP = sum(-log(OR) x log(p\_value)) for ASV with OR>1 and WN = sum(-log(OR) x log(p\_value)) for ASV with OR<1. WTR should be around 1 in case of no tranfer, and larger when present, but due to the high sparsity, the null distribution is not always centered around 1. To calculate the significance of any WTR, the dyads are scrampled to construct a null distribution for the ratio and then compared to the model ratio to calculate a p value.

### 3.2.1 - OVERALL ratio between positive and negative odds

#### 3.2.1.1 - WTR Calculation

**RatioStats\_onesided.RData** contains the formatted output of this section.

#### 3.2.1.2 - WTR tables and figures

Weigthed Transfer Odds as function of delivery mode, compartment and age

|  |  | Models\* | | | Permutation stats† | | |  |
| --- | --- | --- | --- | --- | --- | --- | --- | --- |
| Delivery | Time (days) | ≥1 | <1 | Weigthed ratio | p-value | SE | Null distribution | WTR‡ |
| **Airways** | | | | | | | | |
| CS | 7 | 23 | 81 | 1.210 | 0.539 | 0.516 | 1.253 | 0.966 |
| CS | 30 | 32 | 99 | 2.084 | 0.084 | 0.479 | 1.217 | 1.712 |
| CS | 90 | 29 | 123 | 1.262 | 0.529 | 0.463 | 1.293 | 0.976 |
| Vaginal | 7 | 47 | 246 | 2.287 | 0.034 | 0.425 | 1.172 | 1.951 |
| Vaginal | 30 | 65 | 277 | 3.442 | 0.000 | 0.358 | 1.161 | 2.964 |
| Vaginal | 90 | 55 | 309 | 1.603 | 0.094 | 0.322 | 1.076 | 1.490 |
| **Fecal** | | | | | | | | |
| CS | 7 | 41 | 119 | 2.626 | 0.014 | 0.419 | 1.191 | 2.204 |
| CS | 30 | 37 | 144 | 0.927 | 0.778 | 0.408 | 1.206 | 0.768 |
| CS | 300 | 41 | 120 | 1.638 | 0.216 | 0.408 | 1.258 | 1.302 |
| Vaginal | 7 | 86 | 268 | 4.867 | 0.000 | 0.329 | 1.020 | 4.771 |
| Vaginal | 30 | 94 | 301 | 4.489 | 0.000 | 0.308 | 0.977 | 4.595 |
| Vaginal | 300 | 101 | 303 | 2.508 | 0.005 | 0.328 | 1.106 | 2.268 |
|  |
| --- |
| Note: |
| WTR with p-value < 0.05 are highlighted in bold |
| \* Count of models included with OR ≥1 or <1 |
| † Based on 1000 permutations of the model we calculated the p-value (sum(permutation ratio> weighted ratio)/1000), the standard error of log transformed permutation ratios, and the null distribution (mean permutation ratio) |
| ‡ Reported in the study and calculated as weighted ratio/null distribution |

Figure 4: Weighted transfer ratios from vaginal week 36 to the fecal and airway compartments in first year of life stratified on mode of delivery (blue: vaginal birth, red: cesarean section). A ratio above one indicates enrichment of microbial transfer. Error bars reflect standard errors.

Inference for the difference in weigted ratios between sectio- and vaginal born children

|  |  | Permutational WTRVAG/WTRCS\* | |  |
| --- | --- | --- | --- | --- |
| Time (days) | model WTRVAG/WTRCS | median | mean | p-value |
| **Airways** | | | | |
| 7 | 1.89 | 1.29 | 1.49 | 0.21 |
| 30 | 1.65 | 1.40 | 1.59 | 0.36 |
| 90 | 1.27 | 1.06 | 1.19 | 0.35 |
| **Fecal** | | | | |
| 7 | 1.85 | 1.52 | 1.63 | 0.31 |
| 30 | 4.84 | 1.42 | 1.62 | 0.01 |
| 300 | 1.53 | 1.15 | 1.30 | 0.27 |
|  |
| --- |
| Note: |
| Significantly different WTRVAG and WTRCS (p-value < 0.05) are highlighted in bold |
| \* Based on 1000 permutations of the model, showing median and mean value as well as the p-value (sum(permutation ratio> weighted ratio)/1000) |

### 3.2.2 - WTR at order level

#### 3.2.2.1 - Comparing vaginal to sectio delivery

##### 3.2.2.1.1 - Calculations

**OrderRatioSTATs.RData** contains the WTR at order level for vaginal and CS deliveries

##### 3.2.2.1.2 - Output

Number of ASVs for each taxonomic partitioning based on Order across all models

| Order | min | max | total | median |
| --- | --- | --- | --- | --- |
| Clostridiales | 5 | 144 | 647 | 40.5 |
| Lactobacillales | 21 | 49 | 392 | 31.0 |
| Bacteroidales | 7 | 58 | 345 | 25.5 |
| Selenomonadales | 5 | 31 | 206 | 14.5 |
| Betaproteobacteriales | 6 | 24 | 154 | 11.5 |
| Pseudomonadales | 5 | 23 | 145 | 9.5 |
| Corynebacteriales | 2 | 22 | 140 | 11.0 |
| Bifidobacteriales | 5 | 12 | 103 | 8.0 |
| Enterobacteriales | 5 | 15 | 103 | 8.5 |
| Bacillales | 4 | 17 | 98 | 6.5 |

WTR and statistics for testable orders

|  |  |  | Models\* | | | Permutation stats† | | |  |
| --- | --- | --- | --- | --- | --- | --- | --- | --- | --- |
| Time (days) | Type | Delivery | ≥1 | <1 | Weigthed ratio | p-value | SE | Null distribution | WTR‡ |
| **Bacillales** | | | | | | | | | |
| 7 | Airways | CS | 2 | 5 | 2.498 | 0.151 | 3.351 | 0.645 | 3.872 |
| 7 | Airways | Vaginal | 1 | 16 | 3.096 | 0.258 | 3.761 | 0.398 | 7.770 |
| 7 | Fecal | CS | 1 | 5 | 0.099 | 0.422 | 4.232 | 0.099 | 1.000 |
| 7 | Fecal | Vaginal | 1 | 9 | 0.013 | 0.697 | 4.207 | 0.986 | 0.013 |
| 30 | Airways | CS | 2 | 4 | 5.414 | 0.059 | 2.701 | 4.540 | 1.193 |
| 30 | Airways | Vaginal | 1 | 13 | 0.892 | 0.559 | 2.902 | 1.238 | 0.721 |
| 30 | Fecal | CS | 1 | 5 | 0.525 | 0.470 | 4.204 | 0.525 | 1.000 |
| 30 | Fecal | Vaginal | 1 | 4 | 0.034 | 0.756 | 3.639 | 0.384 | 0.089 |
| 90 | Airways | CS | 2 | 5 | 1.474 | 0.377 | 2.836 | 1.474 | 1.000 |
| 90 | Airways | Vaginal | 1 | 11 | 3.866 | 0.213 | 3.191 | 0.633 | 6.106 |
| 300 | Fecal | CS | 1 | 3 | 0.699 | 0.291 | 4.797 | 0.215 | 3.247 |
| 300 | Fecal | Vaginal | 1 | 3 | 0.279 | 0.508 | 3.810 | 0.298 | 0.936 |
| **Bacteroidales** | | | | | | | | | |
| 7 | Airways | CS | 1 | 6 | 4.305 | 0.159 | 5.387 | 0.001 | 5146.933 |
| 7 | Airways | Vaginal | 2 | 14 | 1.971 | 0.324 | 3.630 | 1.143 | 1.724 |
| 7 | Fecal | CS | 5 | 11 | 1.943 | 0.421 | 1.940 | 1.561 | 1.245 |
| 7 | Fecal | Vaginal | 11 | 37 | 3.141 | 0.048 | 0.904 | 0.978 | 3.212 |
| 30 | Airways | CS | 1 | 9 | 0.752 | 0.469 | 3.884 | 0.752 | 1.000 |
| 30 | Airways | Vaginal | 8 | 21 | 10.037 | 0.006 | 1.887 | 0.965 | 10.400 |
| 30 | Fecal | CS | 4 | 21 | 0.771 | 0.782 | 1.422 | 1.576 | 0.489 |
| 30 | Fecal | Vaginal | 16 | 31 | 8.112 | 0.000 | 0.886 | 0.779 | 10.415 |
| 90 | Airways | CS | 2 | 11 | 2.586 | 0.229 | 2.769 | 0.822 | 3.144 |
| 90 | Airways | Vaginal | 5 | 45 | 0.247 | 0.881 | 1.177 | 0.920 | 0.268 |
| 300 | Fecal | CS | 3 | 23 | 0.406 | 0.935 | 0.857 | 1.337 | 0.304 |
| 300 | Fecal | Vaginal | 17 | 41 | 2.445 | 0.056 | 0.661 | 0.965 | 2.534 |
| **Betaproteobacteriales** | | | | | | | | | |
| 7 | Airways | CS | 1 | 7 | 0.014 | 0.902 | 2.925 | 0.971 | 0.014 |
| 7 | Airways | Vaginal | 3 | 18 | 0.456 | 0.623 | 2.583 | 1.004 | 0.454 |
| 7 | Fecal | CS | 1 | 7 | 0.178 | 0.439 | 4.577 | 0.178 | 1.000 |
| 7 | Fecal | Vaginal | 3 | 13 | 12.047 | 0.015 | 3.149 | 0.566 | 21.299 |
| 30 | Airways | CS | 1 | 6 | 0.003 | 0.813 | 3.645 | 0.564 | 0.005 |
| 30 | Airways | Vaginal | 3 | 18 | 0.710 | 0.543 | 2.623 | 0.860 | 0.825 |
| 30 | Fecal | CS | 0 | 6 | 0.000 | 0.644 | 4.694 | 0.278 | 0.002 |
| 30 | Fecal | Vaginal | 3 | 11 | 6.114 | 0.042 | 3.090 | 0.385 | 15.891 |
| 90 | Airways | CS | 1 | 9 | 0.005 | 0.873 | 3.126 | 0.721 | 0.007 |
| 90 | Airways | Vaginal | 4 | 20 | 0.916 | 0.415 | 2.126 | 0.623 | 1.470 |
| 300 | Fecal | CS | 0 | 6 | 0.000 | 0.620 | 4.773 | 0.406 | 0.001 |
| 300 | Fecal | Vaginal | 3 | 10 | 5.949 | 0.048 | 2.746 | 0.604 | 9.856 |
| **Bifidobacteriales** | | | | | | | | | |
| 7 | Airways | CS | 3 | 2 | 5.738 | 0.145 | 3.188 | 1.087 | 5.280 |
| 7 | Airways | Vaginal | 2 | 7 | 6.283 | 0.142 | 3.301 | 0.595 | 10.553 |
| 7 | Fecal | CS | 2 | 4 | 0.524 | 0.647 | 2.441 | 0.887 | 0.591 |
| 7 | Fecal | Vaginal | 7 | 5 | 7.699 | 0.052 | 1.659 | 0.928 | 8.299 |
| 30 | Airways | CS | 1 | 6 | 0.462 | 0.550 | 4.337 | 0.842 | 0.549 |
| 30 | Airways | Vaginal | 4 | 7 | 5.236 | 0.081 | 2.856 | 0.854 | 6.128 |
| 30 | Fecal | CS | 4 | 4 | 0.782 | 0.575 | 1.632 | 0.994 | 0.787 |
| 30 | Fecal | Vaginal | 6 | 6 | 2.479 | 0.186 | 1.598 | 0.729 | 3.399 |
| 90 | Airways | CS | 2 | 4 | 2.379 | 0.245 | 3.448 | 0.835 | 2.847 |
| 90 | Airways | Vaginal | 3 | 5 | 3.439 | 0.170 | 3.225 | 0.707 | 4.862 |
| 300 | Fecal | CS | 3 | 5 | 0.927 | 0.550 | 1.858 | 1.056 | 0.878 |
| 300 | Fecal | Vaginal | 5 | 6 | 0.437 | 0.668 | 1.711 | 0.951 | 0.460 |
| **Clostridiales** | | | | | | | | | |
| 7 | Airways | CS | 1 | 4 | 0.129 | 0.604 | 3.557 | 0.703 | 0.183 |
| 7 | Airways | Vaginal | 5 | 31 | 4.257 | 0.125 | 2.426 | 1.447 | 2.943 |
| 7 | Fecal | CS | 6 | 32 | 1.881 | 0.224 | 1.196 | 0.941 | 1.999 |
| 7 | Fecal | Vaginal | 14 | 79 | 1.304 | 0.325 | 0.744 | 0.979 | 1.332 |
| 30 | Airways | CS | 3 | 14 | 2.374 | 0.265 | 3.108 | 0.858 | 2.767 |
| 30 | Airways | Vaginal | 9 | 38 | 4.584 | 0.058 | 1.696 | 1.345 | 3.408 |
| 30 | Fecal | CS | 4 | 36 | 0.437 | 0.826 | 0.964 | 1.098 | 0.398 |
| 30 | Fecal | Vaginal | 14 | 95 | 0.600 | 0.831 | 0.592 | 1.099 | 0.546 |
| 90 | Airways | CS | 4 | 15 | 3.305 | 0.176 | 2.436 | 1.373 | 2.406 |
| 90 | Airways | Vaginal | 4 | 54 | 1.284 | 0.444 | 1.017 | 1.158 | 1.109 |
| 300 | Fecal | CS | 18 | 23 | 5.893 | 0.000 | 0.735 | 1.239 | 4.756 |
| 300 | Fecal | Vaginal | 41 | 103 | 3.690 | 0.013 | 0.559 | 1.192 | 3.097 |
| **Corynebacteriales** | | | | | | | | | |
| 7 | Airways | CS | 2 | 7 | 3.371 | 0.243 | 3.384 | 0.900 | 3.744 |
| 7 | Airways | Vaginal | 4 | 17 | 4.323 | 0.086 | 2.521 | 0.739 | 5.851 |
| 7 | Fecal | CS | 2 | 6 | 0.725 | 0.538 | 3.450 | 0.833 | 0.870 |
| 7 | Fecal | Vaginal | 3 | 8 | 4.288 | 0.102 | 2.526 | 0.563 | 7.616 |
| 30 | Airways | CS | 2 | 9 | 0.741 | 0.543 | 3.527 | 1.467 | 0.505 |
| 30 | Airways | Vaginal | 3 | 19 | 0.857 | 0.451 | 1.975 | 0.709 | 1.210 |
| 30 | Fecal | CS | 3 | 3 | 4.250 | 0.110 | 3.239 | 0.492 | 8.645 |
| 30 | Fecal | Vaginal | 4 | 8 | 1.652 | 0.295 | 2.620 | 0.600 | 2.755 |
| 90 | Airways | CS | 1 | 11 | 0.112 | 0.764 | 3.766 | 1.480 | 0.076 |
| 90 | Airways | Vaginal | 2 | 19 | 1.800 | 0.365 | 2.753 | 0.997 | 1.806 |
| 300 | Fecal | CS | 0 | 2 | 0.002 | 0.263 | 5.803 | 0.002 | 1.000 |
| 300 | Fecal | Vaginal | 2 | 3 | 2.716 | 0.213 | 4.757 | 0.333 | 8.153 |
| **Enterobacteriales** | | | | | | | | | |
| 7 | Airways | CS | 1 | 4 | 1.515 | 0.339 | 4.247 | 0.268 | 5.644 |
| 7 | Airways | Vaginal | 4 | 6 | 5.423 | 0.101 | 3.506 | 0.477 | 11.363 |
| 7 | Fecal | CS | 2 | 5 | 12.677 | 0.061 | 3.332 | 1.001 | 12.669 |
| 7 | Fecal | Vaginal | 6 | 9 | 20.863 | 0.002 | 2.318 | 0.615 | 33.947 |
| 30 | Airways | CS | 2 | 3 | 14.357 | 0.056 | 4.188 | 0.430 | 33.381 |
| 30 | Airways | Vaginal | 4 | 4 | 38.543 | 0.007 | 3.222 | 0.345 | 111.880 |
| 30 | Fecal | CS | 1 | 9 | 0.325 | 0.661 | 3.523 | 0.825 | 0.394 |
| 30 | Fecal | Vaginal | 7 | 4 | 37.443 | 0.002 | 2.557 | 0.616 | 60.780 |
| 90 | Airways | CS | 2 | 4 | 3.857 | 0.198 | 3.817 | 0.495 | 7.784 |
| 90 | Airways | Vaginal | 2 | 7 | 13.791 | 0.018 | 3.332 | 0.368 | 37.454 |
| 300 | Fecal | CS | 4 | 3 | 7.967 | 0.105 | 2.969 | 0.771 | 10.334 |
| 300 | Fecal | Vaginal | 5 | 5 | 14.351 | 0.020 | 2.474 | 0.874 | 16.422 |
| **Lactobacillales** | | | | | | | | | |
| 7 | Airways | CS | 1 | 20 | 0.003 | 0.998 | 1.305 | 1.421 | 0.002 |
| 7 | Airways | Vaginal | 7 | 38 | 2.038 | 0.260 | 0.924 | 1.195 | 1.705 |
| 7 | Fecal | CS | 8 | 16 | 4.863 | 0.039 | 0.985 | 0.994 | 4.892 |
| 7 | Fecal | Vaginal | 18 | 24 | 14.754 | 0.000 | 0.730 | 1.059 | 13.933 |
| 30 | Airways | CS | 11 | 13 | 4.006 | 0.051 | 0.945 | 0.967 | 4.141 |
| 30 | Airways | Vaginal | 14 | 35 | 9.293 | 0.003 | 0.866 | 1.363 | 6.819 |
| 30 | Fecal | CS | 6 | 20 | 1.771 | 0.348 | 1.040 | 1.203 | 1.472 |
| 30 | Fecal | Vaginal | 18 | 26 | 18.944 | 0.000 | 0.826 | 1.086 | 17.448 |
| 90 | Airways | CS | 4 | 19 | 0.491 | 0.802 | 1.097 | 1.160 | 0.423 |
| 90 | Airways | Vaginal | 10 | 26 | 7.564 | 0.021 | 1.104 | 1.560 | 4.850 |
| 300 | Fecal | CS | 6 | 16 | 0.577 | 0.746 | 1.174 | 1.197 | 0.482 |
| 300 | Fecal | Vaginal | 6 | 30 | 1.642 | 0.407 | 0.959 | 1.329 | 1.236 |
| **Pseudomonadales** | | | | | | | | | |
| 7 | Airways | CS | 2 | 5 | 3.702 | 0.241 | 3.966 | 1.335 | 2.772 |
| 7 | Airways | Vaginal | 4 | 19 | 1.432 | 0.213 | 2.055 | 0.518 | 2.763 |
| 7 | Fecal | CS | 1 | 5 | 0.404 | 0.558 | 4.124 | 0.608 | 0.664 |
| 7 | Fecal | Vaginal | 3 | 11 | 0.888 | 0.416 | 3.054 | 0.680 | 1.306 |
| 30 | Airways | CS | 1 | 7 | 1.692 | 0.354 | 4.376 | 1.692 | 1.000 |
| 30 | Airways | Vaginal | 4 | 18 | 2.366 | 0.095 | 1.755 | 0.537 | 4.408 |
| 30 | Fecal | CS | 1 | 5 | 0.261 | 0.511 | 4.286 | 0.573 | 0.456 |
| 30 | Fecal | Vaginal | 0 | 12 | 0.000 | 0.957 | 2.916 | 0.476 | 0.001 |
| 90 | Airways | CS | 0 | 9 | 0.000 | 0.862 | 3.119 | 1.282 | 0.000 |
| 90 | Airways | Vaginal | 4 | 19 | 0.115 | 0.860 | 1.627 | 0.584 | 0.197 |
| 300 | Fecal | CS | 0 | 5 | 0.000 | 0.897 | 4.408 | 0.292 | 0.002 |
| 300 | Fecal | Vaginal | 0 | 10 | 0.000 | 0.996 | 4.111 | 0.372 | 0.001 |
| **Selenomonadales** | | | | | | | | | |
| 7 | Airways | CS | 2 | 3 | 2.247 | 0.478 | 4.141 | 1.954 | 1.150 |
| 7 | Airways | Vaginal | 4 | 11 | 5.295 | 0.059 | 2.387 | 0.747 | 7.087 |
| 7 | Fecal | CS | 7 | 5 | 6.460 | 0.027 | 1.769 | 1.046 | 6.177 |
| 7 | Fecal | Vaginal | 11 | 14 | 19.712 | 0.000 | 1.284 | 0.788 | 25.020 |
| 30 | Airways | CS | 1 | 5 | 0.250 | 0.409 | 4.468 | 0.250 | 1.000 |
| 30 | Airways | Vaginal | 3 | 18 | 0.801 | 0.502 | 1.697 | 0.807 | 0.992 |
| 30 | Fecal | CS | 5 | 9 | 1.002 | 0.460 | 1.861 | 0.912 | 1.099 |
| 30 | Fecal | Vaginal | 6 | 24 | 2.662 | 0.105 | 1.362 | 0.713 | 3.733 |
| 90 | Airways | CS | 1 | 9 | 0.599 | 0.682 | 3.127 | 1.095 | 0.547 |
| 90 | Airways | Vaginal | 4 | 20 | 0.406 | 0.661 | 1.355 | 0.642 | 0.633 |
| 300 | Fecal | CS | 2 | 11 | 0.342 | 0.772 | 1.828 | 0.859 | 0.398 |
| 300 | Fecal | Vaginal | 7 | 24 | 3.416 | 0.084 | 1.282 | 0.926 | 3.691 |
|  |
| --- |
| Note: |
| WTR with p-value < 0.05 are highlighted in bold |
| \* Count of models included with OR ≥1 or <1, and the model weighted ratio |
| † Based on 1000 permutations of the model we calculated the p-value (sum(permutation ratio> weighted ratio)/1000), the standard error of log transformed permutation ratios, and the null distribution (mean permutation ratio) |
| ‡ Reported in the study and calculated as weighted ratio/null distribution. For plots this value is truncated so values lower than 0.625 are plotted as 0.625 and values higher than 16 are plotted as 16. |

Figure 5: Weighted transfer ratios (WTRs) from vaginal week 36 to the fecal and airway compartments in the first year of life according to the mode of delivery (blue: vaginal birth, red: cesarean section) partitioned for the 10 most represented taxonomic classes at order level with upper left (Clostridiales) being the most represented order followed by Lactobacillales and so forth. Dashed lines represent analysis on less than 15 ASVs on average. Error bars reflect standard errors. WTR is truncated so values lower than 0.625 are plotted as 0.625 and values higher than 16 are plotted as 16.

#### 3.2.2.2 - Comparing vaginal to scheduled and in labor sectio delivery

##### 3.2.2.2.1 - Calculations

**OrderRatioSTATs\_split.RData** contains the WTR at order level when sectio is split into scheduled and in labor sectio

##### 3.2.2.2.2 - Output

Number of ASVs for each taxonomic partitioning based on Order across all models

| Order | min | max | total | median |
| --- | --- | --- | --- | --- |
| Bacteroidales | 2 | 58 | 353 | 13.0 |
| Betaproteobacteriales | 3 | 24 | 173 | 6.5 |
| Clostridiales | 2 | 144 | 670 | 24.0 |
| Enterobacteriales | 2 | 15 | 112 | 5.0 |
| Lactobacillales | 11 | 49 | 431 | 16.5 |
| Selenomonadales | 3 | 31 | 219 | 9.0 |

WTR and statistics for testable orders

|  |  |  | Models\* | | | Permutation stats† | | |  |
| --- | --- | --- | --- | --- | --- | --- | --- | --- | --- |
| Time (days) | Type | Delivery | ≥1 | <1 | Weigthed ratio | p-value | SE | Null distribution | WTR‡ |
| **Bacteroidales** | | | | | | | | | |
| 7 | Airways | In labor CS | 1 | 3 | 9.357 | 0.030 | 5.588 | 0.001 | 8329.962 |
| 7 | Airways | Scheduled CS | 0 | 3 | 0.002 | 0.214 | 5.532 | 0.002 | 1.000 |
| 7 | Airways | Vaginal | 2 | 14 | 1.971 | 0.324 | 3.630 | 1.143 | 1.724 |
| 7 | Fecal | In labor CS | 4 | 10 | 1.208 | 0.545 | 1.888 | 1.343 | 0.899 |
| 7 | Fecal | Scheduled CS | 1 | 1 | 2.577 | 0.616 | 5.120 | 5.678 | 0.454 |
| 7 | Fecal | Vaginal | 11 | 37 | 3.141 | 0.048 | 0.904 | 0.978 | 3.212 |
| 30 | Airways | In labor CS | 1 | 7 | 0.626 | 0.458 | 3.955 | 0.626 | 1.000 |
| 30 | Airways | Scheduled CS | 0 | 5 | 0.002 | 0.257 | 5.661 | 0.002 | 1.000 |
| 30 | Airways | Vaginal | 8 | 21 | 10.037 | 0.006 | 1.887 | 0.965 | 10.400 |
| 30 | Fecal | In labor CS | 5 | 11 | 2.659 | 0.250 | 1.757 | 1.351 | 1.968 |
| 30 | Fecal | Scheduled CS | 1 | 9 | 0.011 | 0.852 | 3.260 | 1.468 | 0.008 |
| 30 | Fecal | Vaginal | 16 | 31 | 8.112 | 0.000 | 0.886 | 0.779 | 10.415 |
| 90 | Airways | In labor CS | 3 | 7 | 4.385 | 0.224 | 3.088 | 1.297 | 3.382 |
| 90 | Airways | Scheduled CS | 1 | 4 | 1.085 | 0.538 | 4.783 | 2.164 | 0.501 |
| 90 | Airways | Vaginal | 5 | 45 | 0.247 | 0.881 | 1.177 | 0.920 | 0.268 |
| 300 | Fecal | In labor CS | 3 | 9 | 0.301 | 0.923 | 1.140 | 1.179 | 0.255 |
| 300 | Fecal | Scheduled CS | 2 | 14 | 0.742 | 0.718 | 1.580 | 1.201 | 0.618 |
| 300 | Fecal | Vaginal | 17 | 41 | 2.445 | 0.056 | 0.661 | 0.965 | 2.534 |
| **Betaproteobacteriales** | | | | | | | | | |
| 7 | Airways | In labor CS | 1 | 5 | 0.014 | 0.772 | 3.739 | 0.804 | 0.018 |
| 7 | Airways | Scheduled CS | 1 | 4 | 0.027 | 0.731 | 3.443 | 1.710 | 0.016 |
| 7 | Airways | Vaginal | 3 | 18 | 0.456 | 0.623 | 2.583 | 1.004 | 0.454 |
| 7 | Fecal | In labor CS | 0 | 7 | 0.001 | 0.629 | 4.791 | 0.484 | 0.001 |
| 7 | Fecal | Scheduled CS | 1 | 2 | 4.014 | 0.121 | 4.762 | 0.066 | 60.803 |
| 7 | Fecal | Vaginal | 3 | 13 | 12.047 | 0.015 | 3.149 | 0.566 | 21.299 |
| 30 | Airways | In labor CS | 1 | 5 | 0.282 | 0.462 | 3.699 | 0.282 | 1.000 |
| 30 | Airways | Scheduled CS | 0 | 5 | 0.001 | 0.735 | 4.262 | 2.470 | 0.000 |
| 30 | Airways | Vaginal | 3 | 18 | 0.710 | 0.543 | 2.623 | 0.860 | 0.825 |
| 30 | Fecal | In labor CS | 0 | 5 | 0.001 | 0.396 | 5.455 | 0.001 | 1.000 |
| 30 | Fecal | Scheduled CS | 0 | 3 | 0.001 | 0.514 | 5.211 | 1.046 | 0.001 |
| 30 | Fecal | Vaginal | 3 | 11 | 6.114 | 0.042 | 3.090 | 0.385 | 15.891 |
| 90 | Airways | In labor CS | 0 | 8 | 0.001 | 0.811 | 3.786 | 1.648 | 0.000 |
| 90 | Airways | Scheduled CS | 1 | 6 | 0.040 | 0.786 | 3.125 | 1.419 | 0.028 |
| 90 | Airways | Vaginal | 4 | 20 | 0.916 | 0.415 | 2.126 | 0.623 | 1.470 |
| 300 | Fecal | In labor CS | 0 | 5 | 0.001 | 0.326 | 5.597 | 0.001 | 1.000 |
| 300 | Fecal | Scheduled CS | 0 | 4 | 0.001 | 0.491 | 5.257 | 0.001 | 1.000 |
| 300 | Fecal | Vaginal | 3 | 10 | 5.949 | 0.048 | 2.746 | 0.604 | 9.856 |
| **Clostridiales** | | | | | | | | | |
| 7 | Airways | In labor CS | 0 | 4 | 0.001 | 0.619 | 4.840 | 1.935 | 0.000 |
| 7 | Airways | Scheduled CS | 1 | 1 | 0.775 | 0.455 | 5.662 | 0.775 | 1.000 |
| 7 | Airways | Vaginal | 5 | 31 | 4.257 | 0.125 | 2.426 | 1.447 | 2.943 |
| 7 | Fecal | In labor CS | 4 | 14 | 0.691 | 0.646 | 2.051 | 1.111 | 0.622 |
| 7 | Fecal | Scheduled CS | 4 | 22 | 2.688 | 0.237 | 1.435 | 1.264 | 2.126 |
| 7 | Fecal | Vaginal | 14 | 79 | 1.304 | 0.325 | 0.744 | 0.979 | 1.332 |
| 30 | Airways | In labor CS | 2 | 4 | 3.628 | 0.232 | 3.969 | 0.822 | 4.411 |
| 30 | Airways | Scheduled CS | 1 | 10 | 0.437 | 0.341 | 5.110 | 0.437 | 1.000 |
| 30 | Airways | Vaginal | 9 | 38 | 4.584 | 0.058 | 1.696 | 1.345 | 3.408 |
| 30 | Fecal | In labor CS | 4 | 20 | 1.145 | 0.485 | 1.756 | 1.108 | 1.033 |
| 30 | Fecal | Scheduled CS | 2 | 24 | 0.448 | 0.822 | 1.676 | 1.370 | 0.327 |
| 30 | Fecal | Vaginal | 14 | 95 | 0.600 | 0.831 | 0.592 | 1.099 | 0.546 |
| 90 | Airways | In labor CS | 1 | 11 | 1.301 | 0.573 | 3.520 | 1.520 | 0.856 |
| 90 | Airways | Scheduled CS | 3 | 8 | 3.155 | 0.253 | 3.892 | 1.220 | 2.585 |
| 90 | Airways | Vaginal | 4 | 54 | 1.284 | 0.444 | 1.017 | 1.158 | 1.109 |
| 300 | Fecal | In labor CS | 12 | 12 | 8.456 | 0.009 | 1.129 | 1.172 | 7.217 |
| 300 | Fecal | Scheduled CS | 9 | 10 | 2.613 | 0.156 | 0.943 | 1.192 | 2.192 |
| 300 | Fecal | Vaginal | 41 | 103 | 3.690 | 0.013 | 0.559 | 1.192 | 3.097 |
| **Enterobacteriales** | | | | | | | | | |
| 7 | Airways | In labor CS | 2 | 3 | 2.297 | 0.260 | 4.174 | 0.589 | 3.898 |
| 7 | Airways | Scheduled CS | 0 | 2 | 0.001 | 0.824 | 4.530 | 0.068 | 0.021 |
| 7 | Airways | Vaginal | 4 | 6 | 5.423 | 0.101 | 3.506 | 0.477 | 11.363 |
| 7 | Fecal | In labor CS | 3 | 4 | 19.758 | 0.035 | 2.669 | 1.542 | 12.811 |
| 7 | Fecal | Scheduled CS | 1 | 2 | 2.329 | 0.593 | 4.076 | 3.192 | 0.730 |
| 7 | Fecal | Vaginal | 6 | 9 | 20.863 | 0.002 | 2.318 | 0.615 | 33.947 |
| 30 | Airways | In labor CS | 3 | 1 | 15.075 | 0.066 | 3.925 | 0.678 | 22.243 |
| 30 | Airways | Scheduled CS | 0 | 2 | 0.002 | 0.748 | 4.945 | 0.157 | 0.012 |
| 30 | Airways | Vaginal | 4 | 4 | 38.543 | 0.007 | 3.222 | 0.345 | 111.880 |
| 30 | Fecal | In labor CS | 1 | 4 | 0.631 | 0.545 | 3.952 | 0.707 | 0.893 |
| 30 | Fecal | Scheduled CS | 1 | 3 | 0.150 | 0.831 | 3.651 | 1.008 | 0.149 |
| 30 | Fecal | Vaginal | 7 | 4 | 37.443 | 0.002 | 2.557 | 0.616 | 60.780 |
| 90 | Airways | In labor CS | 1 | 3 | 6.447 | 0.078 | 4.410 | 0.278 | 23.176 |
| 90 | Airways | Scheduled CS | 1 | 3 | 1.074 | 0.356 | 4.665 | 0.119 | 9.026 |
| 90 | Airways | Vaginal | 2 | 7 | 13.791 | 0.018 | 3.332 | 0.368 | 37.454 |
| 300 | Fecal | In labor CS | 4 | 2 | 11.455 | 0.070 | 3.063 | 0.703 | 16.301 |
| 300 | Fecal | Scheduled CS | 1 | 2 | 2.856 | 0.258 | 5.063 | 2.856 | 1.000 |
| 300 | Fecal | Vaginal | 5 | 5 | 14.351 | 0.020 | 2.474 | 0.874 | 16.422 |
| **Lactobacillales** | | | | | | | | | |
| 7 | Airways | In labor CS | 1 | 12 | 0.177 | 0.915 | 1.634 | 1.029 | 0.172 |
| 7 | Airways | Scheduled CS | 1 | 10 | 0.008 | 0.987 | 1.459 | 1.174 | 0.007 |
| 7 | Airways | Vaginal | 7 | 38 | 2.038 | 0.260 | 0.924 | 1.195 | 1.705 |
| 7 | Fecal | In labor CS | 8 | 10 | 4.576 | 0.077 | 1.131 | 1.202 | 3.806 |
| 7 | Fecal | Scheduled CS | 5 | 10 | 2.432 | 0.214 | 1.368 | 0.946 | 2.571 |
| 7 | Fecal | Vaginal | 18 | 24 | 14.754 | 0.000 | 0.730 | 1.059 | 13.933 |
| 30 | Airways | In labor CS | 9 | 8 | 4.883 | 0.027 | 0.948 | 0.859 | 5.684 |
| 30 | Airways | Scheduled CS | 4 | 9 | 1.945 | 0.286 | 1.542 | 0.947 | 2.053 |
| 30 | Airways | Vaginal | 14 | 35 | 9.293 | 0.003 | 0.866 | 1.363 | 6.819 |
| 30 | Fecal | In labor CS | 8 | 12 | 2.597 | 0.182 | 1.177 | 1.023 | 2.540 |
| 30 | Fecal | Scheduled CS | 4 | 12 | 0.919 | 0.632 | 1.435 | 1.310 | 0.701 |
| 30 | Fecal | Vaginal | 18 | 26 | 18.944 | 0.000 | 0.826 | 1.086 | 17.448 |
| 90 | Airways | In labor CS | 5 | 11 | 0.466 | 0.786 | 0.917 | 0.959 | 0.486 |
| 90 | Airways | Scheduled CS | 3 | 9 | 1.749 | 0.386 | 1.682 | 1.252 | 1.396 |
| 90 | Airways | Vaginal | 10 | 26 | 7.564 | 0.021 | 1.104 | 1.560 | 4.850 |
| 300 | Fecal | In labor CS | 5 | 9 | 2.256 | 0.361 | 1.727 | 1.431 | 1.577 |
| 300 | Fecal | Scheduled CS | 4 | 10 | 1.536 | 0.395 | 1.499 | 1.261 | 1.218 |
| 300 | Fecal | Vaginal | 6 | 30 | 1.642 | 0.407 | 0.959 | 1.329 | 1.236 |
| **Selenomonadales** | | | | | | | | | |
| 7 | Airways | In labor CS | 0 | 3 | 0.001 | 0.775 | 4.408 | 0.846 | 0.001 |
| 7 | Airways | Scheduled CS | 2 | 1 | 45.411 | 0.004 | 4.540 | 0.322 | 140.939 |
| 7 | Airways | Vaginal | 4 | 11 | 5.295 | 0.059 | 2.387 | 0.747 | 7.087 |
| 7 | Fecal | In labor CS | 5 | 1 | 110.383 | 0.001 | 3.184 | 1.297 | 85.119 |
| 7 | Fecal | Scheduled CS | 3 | 3 | 3.372 | 0.194 | 2.869 | 0.917 | 3.677 |
| 7 | Fecal | Vaginal | 11 | 14 | 19.712 | 0.000 | 1.284 | 0.788 | 25.020 |
| 30 | Airways | In labor CS | 1 | 2 | 1.046 | 0.220 | 5.156 | 0.002 | 418.577 |
| 30 | Airways | Scheduled CS | 1 | 3 | 0.207 | 0.361 | 4.290 | 0.207 | 1.000 |
| 30 | Airways | Vaginal | 3 | 18 | 0.801 | 0.502 | 1.697 | 0.807 | 0.992 |
| 30 | Fecal | In labor CS | 5 | 4 | 4.816 | 0.098 | 2.122 | 1.059 | 4.547 |
| 30 | Fecal | Scheduled CS | 2 | 8 | 0.919 | 0.581 | 2.373 | 1.238 | 0.743 |
| 30 | Fecal | Vaginal | 6 | 24 | 2.662 | 0.105 | 1.362 | 0.713 | 3.733 |
| 90 | Airways | In labor CS | 1 | 5 | 0.681 | 0.685 | 3.388 | 1.518 | 0.448 |
| 90 | Airways | Scheduled CS | 0 | 5 | 0.002 | 0.574 | 4.678 | 0.265 | 0.006 |
| 90 | Airways | Vaginal | 4 | 20 | 0.406 | 0.661 | 1.355 | 0.642 | 0.633 |
| 300 | Fecal | In labor CS | 3 | 6 | 1.142 | 0.472 | 2.718 | 1.080 | 1.058 |
| 300 | Fecal | Scheduled CS | 0 | 9 | 0.000 | 0.918 | 2.699 | 1.029 | 0.000 |
| 300 | Fecal | Vaginal | 7 | 24 | 3.416 | 0.084 | 1.282 | 0.926 | 3.691 |
|  |
| --- |
| Note: |
| WTR with p-value < 0.05 are highlighted in bold |
| \* Count of models included with OR ≥1 or <1, and the model weighted ratio |
| † Based on 1000 permutations of the model we calculated the p-value (sum(permutation ratio> weighted ratio)/1000), the standard error of log transformed permutation ratios, and the null distribution (mean permutation ratio) |
| ‡ Reported in the study and calculated as weighted ratio/null distribution. For plots this value is truncated to be between 0.625 and 16 |

Figure 5-figure supplement 1: Weighted transfer ratios (WTRs) from vaginal week 36 to the fecal and airway compartments in the first year of life according to the mode of delivery (blue: vaginal birth, red: in labor CS, green: scheduled CS) partitioned for the 10 most represented taxonomic classes at order level with upper left (Clostridiales) being the most represented family followed by Lactobacillales and so forth. Dashed lines represent analysis on less than 15 ASVs on average. Error bars reflect standard errors. WTR is truncated so values lower than 0.625 are plotted as 0.625 and values higher than 16 are plotted as 16.

## 3.3 - Transfer of mothers dominant ASV

In this analysis, the vaginal dominating ASV in each mother is looked for in the corresponding child. This analysis is ASV *unspecific* I.e. just the dominating one we look for.

### 3.3.1 - Calculations

**Winnerstats.RData** contains the result for transfer of mothers dominant ASVs

### 3.3.2 - Output

Transfer of mosthers most dominating ASVs. The frequency of the ASV in the child is shown on the y-axis, color indicate delivery mode, x-axis the domination rank (1 = most dominating, 2 = second,…), label refers to p-values towards H0 of no transfer.

Plot data

|  |  |  | Samples\* | | |  |
| --- | --- | --- | --- | --- | --- | --- |
| Time | Delivery | Rank | Child | Mother | shared (%) | p-value |
| **Airways** | | | | | | |
| 7 | Sectio | 1 | 9 | 102 | 8.824 | 0.526 |
| 7 | Sectio | 2 | 9 | 102 | 8.824 | 0.132 |
| 7 | Sectio | 3 | 7 | 102 | 6.863 | 0.302 |
| 7 | Sectio | 4 | 4 | 102 | 3.922 | 0.841 |
| 7 | Vaginal | 1 | 56 | 424 | 13.208 | 0.003 |
| 7 | Vaginal | 2 | 24 | 424 | 5.660 | 0.867 |
| 30 | Sectio | 1 | 10 | 126 | 7.937 | 0.493 |
| 30 | Sectio | 2 | 9 | 126 | 7.143 | 0.336 |
| 30 | Sectio | 3 | 9 | 126 | 7.143 | 0.313 |
| 30 | Sectio | 4 | 5 | 126 | 3.968 | 0.839 |
| 30 | Vaginal | 1 | 57 | 480 | 11.875 | 0.003 |
| 30 | Vaginal | 2 | 42 | 480 | 8.750 | 0.012 |
| 90 | Sectio | 1 | 9 | 125 | 7.200 | 0.678 |
| 90 | Sectio | 2 | 6 | 125 | 4.800 | 0.800 |
| 90 | Sectio | 3 | 8 | 125 | 6.400 | 0.406 |
| 90 | Sectio | 4 | 7 | 125 | 5.600 | 0.337 |
| 90 | Vaginal | 1 | 52 | 489 | 10.634 | 0.075 |
| 90 | Vaginal | 2 | 35 | 489 | 7.157 | 0.160 |
| **Fecal** | | | | | | |
| 7 | Sectio | 1 | 17 | 107 | 15.888 | 0.174 |
| 7 | Sectio | 2 | 10 | 107 | 9.346 | 0.561 |
| 7 | Sectio | 3 | 12 | 107 | 11.215 | 0.202 |
| 7 | Sectio | 4 | 11 | 107 | 10.280 | 0.240 |
| 7 | Sectio | 5 | 13 | 107 | 12.150 | 0.126 |
| 7 | Vaginal | 1 | 81 | 426 | 19.014 | 0.000 |
| 7 | Vaginal | 2 | 62 | 426 | 14.554 | 0.015 |
| 30 | Sectio | 1 | 18 | 114 | 15.789 | 0.646 |
| 30 | Sectio | 2 | 17 | 114 | 14.912 | 0.323 |
| 30 | Sectio | 3 | 11 | 114 | 9.649 | 0.482 |
| 30 | Sectio | 4 | 14 | 114 | 12.281 | 0.291 |
| 30 | Vaginal | 1 | 85 | 461 | 18.438 | 0.604 |
| 30 | Vaginal | 2 | 79 | 461 | 17.137 | 0.006 |
| 300 | Sectio | 1 | 15 | 114 | 13.158 | 0.967 |
| 300 | Sectio | 2 | 14 | 114 | 12.281 | 0.171 |
| 300 | Sectio | 3 | 9 | 114 | 7.895 | 0.373 |
| 300 | Sectio | 4 | 7 | 114 | 6.140 | 0.734 |
| 300 | Vaginal | 1 | 91 | 466 | 19.528 | 0.327 |
| 300 | Vaginal | 2 | 52 | 466 | 11.159 | 0.828 |
|  |
| --- |
| \* Count of samples where that rank ASV is present and the percentage of pairs where it is shared |

Ranking of ASVs. I.e. which ASVs are dominating at which rank and in how many mothers does it have that rank

| ASV | Samples (n) |
| --- | --- |
| **Rank 1** | |
| Genus\_Lactobacillus\_139 | 227 |
| Genus\_Lactobacillus\_323 | 224 |
| Lactobacillus\_gasseri\_37 | 76 |
| Genus\_Gardnerella\_40 | 30 |
| Genus\_Gardnerella\_20 | 22 |
| Genus\_Lactobacillus\_268 | 22 |
| **Rank 2** | |
| Genus\_Lactobacillus\_139 | 93 |
| Genus\_Lactobacillus\_268 | 82 |
| Genus\_Lactobacillus\_323 | 70 |
| Lactobacillus\_gasseri\_37 | 59 |
| Genus\_Gardnerella\_40 | 47 |
| Genus\_Gardnerella\_20 | 44 |
| Genus\_Megasphaera\_22 | 17 |
| Genus\_Bifidobacterium\_60 | 15 |
| Lactobacillus\_reuteri\_33 | 15 |
| **Rank 3** | |
| Genus\_Lactobacillus\_139 | 86 |
| Genus\_Lactobacillus\_323 | 73 |
| Lactobacillus\_gasseri\_37 | 47 |
| Genus\_Gardnerella\_20 | 41 |
| Genus\_Gardnerella\_40 | 37 |
| Genus\_Lactobacillus\_268 | 36 |
| Lactobacillus\_reuteri\_33 | 35 |
| Genus\_Megasphaera\_22 | 24 |
| Genus\_Atopobium\_36 | 17 |
| **Rank 4** | |
| Genus\_Lactobacillus\_323 | 70 |
| Lactobacillus\_gasseri\_37 | 60 |
| Genus\_Lactobacillus\_139 | 48 |
| Genus\_Gardnerella\_40 | 45 |
| Genus\_Lactobacillus\_268 | 40 |
| Genus\_Gardnerella\_20 | 36 |
| Lactobacillus\_reuteri\_33 | 32 |
| Genus\_Ureaplasma\_8 | 19 |
| Genus\_Megasphaera\_22 | 15 |

## 3.4 - Phylogenetic tree with transfer odds

Results on the tree of life Here, we have the individual results shown on the phylogenetic tree

Phylogenetic tree with transfer odds
